# Identification of differentially expressed genes and SNPs linked to harvest body weight of genetically improved rohu carp, *Labeo rohita*

**DOI:** 10.3389/fgene.2023.1153911

**Published:** 2023-06-08

**Authors:** P. Nandanpawar, L. Sahoo, B. Sahoo, K. Murmu, A. Chaudhari, A. Pavan kumar, P. Das

**Affiliations:** ^1^ ICAR-Central Institute of Freshwater Aquaculture, Bhubaneswar, Odisha, India; ^2^ ICAR-Central Institute of Fisheries Education, Mumbai, Maharashtra, India

**Keywords:** body weight, mRNA-seq, differentially expressed transcripts, breeding value, *Labeo rohita*

## Abstract

In most of the aquaculture selection programs, harvest body weight has been a preferred performance trait for improvement. Molecular interplay of genes linked to higher body weight is not elucidated in major carp species. The genetically improved rohu carp with 18% average genetic gain per generation with respect to harvest body weight is a promising candidate for studying genes’ underlying performance traits. In the present study, muscle transcriptome sequencing of two groups of individuals, with significant difference in breeding value, belonging to the tenth generation of rohu carp was performed using the Illumina HiSeq 2000 platform. A total of 178 million paired-end raw reads were generated to give rise to 173 million reads after quality control and trimming. The genome-guided transcriptome assembly and differential gene expression produced 11,86,119 transcripts and 451 upregulated and 181 downregulated differentially expressed genes (DEGs) between high-breeding value and low-breeding value (HB & LB) groups, respectively. Similarly, 39,158 high-quality coding SNPs were identified with the Ts/Tv ratio of 1.23. Out of a total of 17 qPCR-validated transcripts, eight were associated with cellular growth and proliferation and harbored 13 SNPs. The gene expression pattern was observed to be positively correlated with RNA-seq data for genes such as myogenic factor 6, titin isoform X11, IGF-1 like, acetyl-CoA, and thyroid receptor hormone beta. A total of 26 miRNA target interactions were also identified to be associated with significant DETs (*p-value* < 0.05). Genes such as Myo6, IGF-1-like, and acetyl-CoA linked to higher harvest body weight may serve as candidate genes in marker-assisted breeding and SNP array construction for genome-wide association studies and genomic selection.

## 1 Introduction

Aquaculture is one of the fastest growing food production sectors providing animal protein at affordable cost. However, the demand for fish is likely to double in the near future due to population growth, increased average income, and urbanization. Aquaculture production is expected to reach 105 Mt by 2029, 10 Mt more than that of the capture sector (OECD & Food and Agriculture Organization of the United Nations, 2022). To meet the demand, several technological interventions like system and species diversification, better management practices, and genetic selection are in use. Genetic improvement through selective breeding has emerged as one of the preferred methods to increase the production and productivity. Successful genetic selection for growth traits has been demonstrated in several fish species (Gjedrem, 2000). As per the estimate derived from those species under selection, the average genetic gain in growth rate is over 12.5% per generation, and the aquaculture production could be rapidly increased if selective breeding is applied to farmed animals (Gjedrem and Robinson, 2014). The genetic gain for growth rates reported through genetic selection for different fish species, for example, are 15% in Atlantic salmon, 13% in rainbow trout, 12%–18% in channel catfish (Gjedrem, 2000; Gjedrem and Robinson, 2014), 15% in tilapia (Nguyen et al., 2010; Nguyen, 2016), and 7% for common carp (Ninh et al., 2011; Dong, Nguyen, and Zhu, 2015), and in aquatic invertebrates, the reported genetic gain rate was 11% for the giant freshwater prawn (Pillai et al., 2022) and 18.4% for the Pacific white shrimp (Castillo-Juárez et al., 2015) with medium-to-high heritability.

India had its first genetic improvement program for rohu carp in 1992 at ICAR-Central Institute of Freshwater Aquaculture (ICAR-CIFA), Bhubaneswar, in collaboration with the Institute of Aquaculture Research (AKVAFORSK), Norway. Under this program, the base population for selective breeding was developed by collecting rohu fingerlings from five major rivers Ganga, Yamuna, Brahmaputra, Sutlej, and Gomati along with the ICAR-CIFA farm stock. The improved rohu, “Jayanti™,” has achieved an average genetic gain of 18% per generation after nine generations of selection (Mahapatra et al., 2017) and heritability estimates of 0.2–0.3 in mono and polyculture ponds (Gjerde et al., 2019). Using the index selection method and BLUP ranking for selection of potential parents, a total of 49 males and 33 females were used to produce 51 families belonging to the tenth generation rohu. After fishes attained taggable size, i.e. 10–15 gms, 40 families were tagged with passive integrated transponder (PIT) tags and stocked in communal rearing ponds. Presently, the twelfth generation of rohu selected for higher growth rate is being maintained along with one control line at ICAR-CIFA. The seed of “Jayanti” is in great demand as it grows significantly better than local rohu (Mahapatra et al., 2017). Selective breeding of rohu for enhanced growth has been successfully demonstrated in India through different hatcheries and fish farmers.

Body weight is a commercially important trait having polygenic control of expression. Muscle growth is dependent on the quantum of hypertrophy and hyperplasia. In fishes, this dynamics functions under strict control of several growth-associated genes such as IGF, GH, and GHR2; myogenic regulatory factors such as MyoD and Myf6 (Liu et al., 2020a); and structural genes such as myosin, myotrophin, and titin (Alves-Costa, Silva, and Wasko, 2015). There have been some reports on systems of controlling harvest body weight of fishes, in which one of them is compensatory growth. It is a period of accelerated growth that occurs when an appropriate environment is provided following a spell of growth depression as a result of prolonged feed scarcity. This system is useful in the aquaculture setup to control body growth according to the harvesting period. Molecular responses upon feed restriction as an effect of compensatory growth includes upregulation of hyperphagic ghrelin and GH-related genes followed by a period of food deprivation that triggers compensatory growth in *Labeo rohita* (Dar et al., 2018; Dar et al., 2020). The hormonal system of controlling body growth includes major hormonal cascades such as the GH–IGF system, ghrelin–leptin system, and thyroid for metabolism and appetite control are well-explained in temperate fishes such as cod and salmon (Weidner et al., 2020) and to some extent in *Labeo rohita* (Dar et al., 2020; Shahjahan et al., 2020; Shahjahan et al., 2021). Gene editing/knockout of muscle growth-related genes has been attempted in channel catfish where a faster growth was observed after knockout of the *myostatin* gene (Yeh et al., 2017; Coogan et al., 2022). However, the underlying biological processes may involve complex gene regulation networks comprising several interacting genes with varying effects.

Integration of genomic tools into selective breeding programs has expedited the pace of genetic improvement with accuracy (Fjalestad, Moen, and Gomez-Raya, 2003; Laghari et al., 2014). This has been possible mostly due to development of high-end genomic resources, particularly, draft genome sequences, and thereby genome-wide SNPs and arrays in a number of non-model fish species such as salmon, catfish, and trout (Coppe et al., 2010; Dominik et al., 2010; Dan et al., 2013; Liu et al., 2014; Sun et al., 2017; Salem et al., 2018; Yoshida et al., 2018; Mastrochirico-Filho et al., 2020) to be implemented in QTL identification marker-assisted selection and/or genomic selection. Furthermore, improved bioinformatics pipelines made it possible to automate the large-scale genotyping of SNPs (Slate et al., 2009), and their subsequent utilization in high-resolution linkage maps and genome-wide association studies (Bennet et al., 2010; Salem et al., 2012; Li et al., 2016; Joshi et al., 2018; Hillestad et al., 2020; Yáñez et al., 2022) has been elucidated. Use of differential expression data from transcriptomes for identification of genes linked to improved growth (Chealoh et al., 2018; Duran et al., 2022; Shen et al., 2022), disease resistance (Yanez, Houston, and Newman, 2014; Tadmor-Levi et al., 2019), and low saline adaptation (Lin et al., 2019; Powell et al., 2021) has been instrumental in understanding molecular pathways associated with performance traits. In rohu, a number of genomics resources have been generated in the last 1 decade to enable genome-guided data analysis and marker-assisted breeding programs (Robinson et al., 2012; Robinson et al., 2014; Das et al., 2020; Arick et al., 2022). However, the genetic architecture underlying harvest body weight in rohu carp has not yet been elucidated.

The present investigation focuses on the molecular underplays linked to harvest body weight of rohu pertaining to the ongoing selective breeding program currently operating in its twelfth generation. It is expected that there might be many genes significantly contributing to the improved growth rate, which is the trait under selection. Considering this, the present work was planned to identify significant differentially expressed genes (DEGs) in high-breeding value (HB) and low-breeding value (LB) groups of improved carp and their possible association with higher body weight. A positive correlation of body growth and disease resistance to the aeromoniasis trait in improved rohu as per earlier reports (Robinson et al., 2012; Robinson et al., 2014; Gjerde et al., 2019) prompted us further to identify DEGs and pathways linked to disease resistance in the HB group. As transcription factors (TFs) have a pivotal role in modulating gene expression profiles, *in silico* TF enrichment analysis of DEGs was performed along with the description of an upstream regulatory kinase network. Furthermore, identification of miRNA targets in selected DEGs was attempted to pinpoint putative miRNAs for possible downstream application in regulation of gene expression. Last, identification of coding SNPs and annotation was performed with the aim to find out their association and possible effects on growth-related gene expression profiles. The information on candidate genes will be useful for marker-assisted breeding, SNP array construction for genome-wide association studies, and genomic selection. Validation of expression dynamics of growth- and immunity-related genes shall aid in visualizing how genetic selection changes the transcript landscape for improving traits under selection. Apart from this, highly enriched miRNA targets and transcription factors shall act as resources for tailored regulation of gene expression in rohu and related species. Furthermore, SNP hotspots associated with DEGs identified through mapping against chromosomes shall serve as putative candidates as QTLs for body growth. Further validation in terms of phenotype contribution shall be instrumental for simultaneous trait selection with respect to correlated traits.

## 2 Materials and methods

### 2.1 Sampling, RNA extraction, and quantification

For this study, muscle tissues were originated from an ongoing selection breeding program for the harvest body weight of rohu carp (Mahapatra et al., 2017). Muscle tissues were collected from six individuals with HB value and five with LB value. Phenotypic data such as length and weight were recorded, and the relationship was calculated using Microsoft Excel (Microsoft Corporation, 2018). Fishes were dissected and 50-mg muscle tissues were chopped with a sterile scalpel and snap-frozen in liquid nitrogen. Handling and sampling of fish was performed following the guidelines for control and supervision of experiments on animals by the Government of India and approved by the Institutional Animal Ethics Committee (IAEC) of ICAR-CIFA. The snap-frozen muscle tissues were homogenized in a tissue lyser for 1 min. The homogenized tissues were added with 1 mL of TRIzol reagent by vortexing to lyse the cells and stabilize the RNA. The homogenate was mixed with an equal volume of absolute ethanol and applied onto the Zymo-Spin column. RNA elution was performed in 20 µl of nuclease-free water. The RNA quality assessment was carried out using the RNA ScreenTape system (Agilent) in a 4150 TapeStation system (Agilent) to calculate the RIN values. RNA concentration was determined on a Qubit 3.0 Fluorometer using the Qubit™ RNA BR Assay Kit (Thermo Fisher Scientific).

### 2.2 mRNA enrichment, library preparation, and sequencing

Following the manufacturer’s protocol, 500 ng total RNA (two individuals/library) was used to enrich the mRNA using the NEBNext Poly(A) mRNA Magnetic Isolation Module (New England Biolabs). The enriched mRNA was used to prepare six libraries (three in the HB group and three in the LB group) with the NEBNext^®^ UltraTM II RNA Library Prep Kit for Illumina (New England Biolabs). The fragmented mRNAs were reverse-transcribed to form cDNA after being primed with NEBNext Random Primers and cleaned with 1.8 X AMPure XP beads (Beckman Coulter). Loop adapters were ligated to the adenylated fragments and cleaved with the uracil-specific excision reagent (USER) enzyme. The cDNA was amplified (10 times) and purified with 0.9 X AMPure beads (Beckman Coulter). Sequencing of libraries was performed using the Illumina HiSeq 2000 platform (PE 2 × 100 bp).

### 2.3 Identification of differentially expressed transcripts, functional annotation enrichment, and validation using qPCR

FastQC (Andrews, 2010) and AfterQC (Chen et al., 2017) were used to visualize quality and remove adapters. Low-quality reads (Q 20), and unpaired reads from Illumina short raw reads along with filtered high-quality reads were subjected to genome-guided assembly using the chromosome-level genome (NCBI Bioproject: PRJNA887821) of *Labeo rohita* in Trinity (ver.2.11) (Grabherr et al., 2011). Benchmarking Universal Single-Copy Ortholog (BUSCO) analysis was performed using the Eukaryota and core vertebrate gene (CVG) datasets (Manni et al., 2021). The contigs were merged according to a similarity criterion of 90% in CD-HIT-EST (version 4.6.3) (Fu et al., 2012). For identification of protein coding regions in a transcriptome, the assembled transcripts were analyzed using TransDecoder (https://github.com/TransDecoder) (Haas et al., 2013).

Simultaneously, high-quality filtered reads from all six libraries were aligned to the rohu reference genome using the genome-guided assembly mode of command line-based software Trinity (ver 2.11) with default parameters. Estimation of N50 and ExN50 using BUSCO ensured the accuracy and effectiveness of the assembly outputs. The BAM mapping files were fed to edgeR (Robinson, McCarthy, and Smyth, 2010) for differential gene expression analysis. The transcripts were considered differentially expressed if |log2 fold change| ≥1 and adjusted *p-value* < 0.5 × 10^−3^ (Benjamini, Heller, and Yekutieli, 2009). Furthermore, transcripts with |log2 fold change| ≥2 were screened, mapped, and annotated using the Blast2Go Pro feature in OmicsBox 2.2 (Conesa et al., 2005) to allocate gene descriptions at an E-value cut-off of 1 × 10^−6^ against the NCBI RefSeq database. These annotated DETs were then mapped to KEGG pathways using the “Load KEGG pathways” in the OmicsBox suite, elucidating the network of pathways involved in contributing differential body weight in fishes under study. Functional classification was accomplished using WEGO software (Ye et al., 2018). A total of 16 pathways of interest were chosen from the allocated KEGG pathways to decipher the involvement of DETs in body growth. The protein–protein interaction (PPI) was executed using the K-means clustering method in the STRING database (https://string-db.org/) (Szklarczyk et al., 2019).

For validation of DETs**,** total RNA was extracted from all the collected samples using TRIzol reagent (Sigma-Aldrich, St. Louis, MO, USA). Synthesis of first-strand cDNA was carried out using the Prime Script™ first-strand cDNA Synthesis kit (DSS TAKARA Bio, Japan) primed with the Oligo dT primer as per the manufacturer’s protocol and stored at −25°C until use. A total of 18 primer pairs (12 upregulated and six downregulated; Supplementary Table S1) were designed using the Primer-BLAST online server (Ye et al., 2012) for transcripts that were differentially expressed in muscle tissues of rohu between the HB and LB groups. The specificity of all primers was tested using conventional PCR, with TM specific to the primer set (Table S1). RT-qPCR was performed in triplicates for muscle tissues of rohu belonging to HB and LB groups (four fishes/group) using the LightCycler 96 SW 1.1 (Roche, Germany). In a 96-well plate, each 20 μl reaction volume contained 2 μl cDNA (10 ng), 0.2 μl of each primer (concentration 10 μM), 5 μl of 2X FastStart Essential DNA Green Master Mix (Roche, Germany), and 12.6 μl Milli-Q water. Conversion of raw data to cycle threshold (Ct) values was achieved using the software provided by the LightCycler 96 SW 1.1 system (Roche, Germany). Quantification of relative gene expression was performed using the delta–delta (2^−ΔΔCT^) method (Livak and Schmittgen, 2001) and using beta actin as the housekeeping gene for normalization of relative expression. For calculation of *p-*values for differentially expressed transcripts between HB and LB groups, the paired *t*-test was used. The fold-change value for each gene was calculated as the expression ratio in the HB group to that in the LB group. All results were correlated with RNA-seq-log2fold change data using Microsoft Excel (Microsoft Corporation, 2018) to examine the trend of gene expression. Significantly expressed (>4 fold) and validated DETs were used to query GO for biological process, GOSlim process, and KEGG pathways using ShinyGO 0.76 (http://bioinformatics.sdstate.edu/go/) (Ge et al., 2020) to know enriched GO terms contributed by them. For enrichment analyses, the default statistical parameters were used. Ranking of statistical significance of terms was performed using hypergeometric and chi-square methods to identify highly tempered biological processes.

### 2.4 Transcription factor prediction and enrichment analysis and prediction of target interactions

To predict transcription factors from assembled transcripts, the animal transcription factor database AnimalTFDB 3.0 (http://bioinfo.life.hust.edu.cn/AnimalTFDB/#!/predict) (H. Hu et al., 2019) was used. Using this information, TFEA was performed on 39 top predicted transcription factors attained from significant DETs using the X2K webserver. In order to find out miRNA targets associated with significant DETs, the *enrichr* web service (https://maayanlab.cloud/Enrichr/) (Chen et al., 2013) was used with miRTarBase as validated miRNA target database and zebrafish as a model organism. miRNA targets associated with selected transcription factors were predicted using GeneCodis 4 (García-Moreno et al., 2021).

### 2.5 Coding SNP discovery and annotation

Filtered reads were subjected to mapping with *Labeo rohita-*indexed chromosome level genome (NCBI Bioproject: PRJNA887821)) in BWA-MEM command line software (Li, 2013) followed by SAM to BAM file conversion and SNP calling in BCFtools (Li, 2013). The sequences were mapped to the reference genome with a maximum of two mismatches in each sequence for the assembly step. The following quality and significance filters were used to detect SNPs: 1) minimum average quality of surrounding bases = 20 quality score units, 2) minimum coverage = 10 reads, and 3) minimum variant frequency or count = 20% or two read counts per SNP. High-quality SNPs were annotated using the “SnpEff” module of the Galaxy webserver (https://usegalaxy.org) (Cingolani et al., 2012). Identification of QTLs associated with SNP harboring DETs was carried out using Animal QTLdb taking the rainbow trout as a model organism (https://www.animalgenome.org/cgi-bin/QTLdb/) (Hu et al., 2013).

## 3 Results

### 3.1 Phenotypic data analysis, mRNA sequencing, QC, and assembly

**FIGURE 1:**
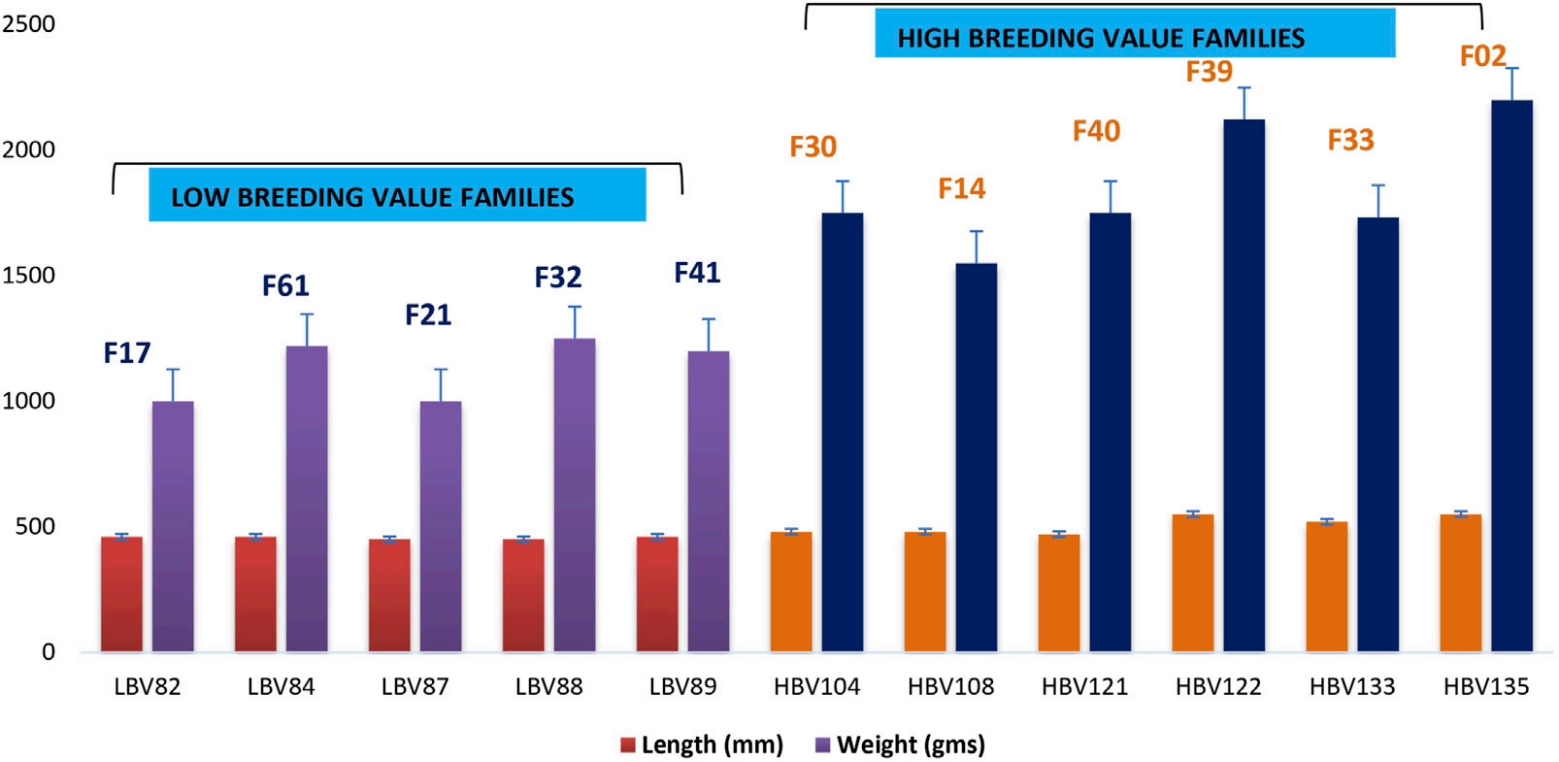
Phenotypic data of adult rohu from LB and HB families along with their EBVs.

Fish belonging to the LB group had a mean length 45.6 cm and weight 1.1 kg, while for the HB group, the mean length was 50.8 cm and weight 1.85 kg (Figure 1). The length–weight relationship analysis indicated “b” value 3.074, which showed that growth was slightly positive allometric. R^2^ value of 0.89 indicated positive association between length and weight (Supplementary Figure S1). Condition factor “K” was found to be 1.07 and indicated the wellbeing of fish under study and its environment.

Sequencing of six NGS libraries derived from good-quality muscle RNA (RIN >6) from both the groups yielded 178 million paired-end reads (2 × 150) (Supplementary Table S2). The data has been deposited in Sequence Read Archive (NCBI) database with Bioproject Accession ID: PRJNA8944. Quality analysis and stringent filtering of low-quality reads resulted in 173 million reads, with GC content ranging from 37% to 42%. The average read length and Phred score were 151 bp and 35, respectively (Table 1). High-quality reads were assembled using high-quality rohu reference genome into 11,86,119 contigs with N50 of 1123 bp. A total of 736,781,229 bases were assembled. BUSCO analysis of assembled contigs revealed 58.74% completeness of the assembly (Table 2). CD-HIT EST analysis revealed 10,97,979 clusters of UniGenes. Identification of protein coding regions in the transcriptome revealed a total of 5,34,791 complete ORFs. As an indicator of annotation performance, the GO-level distribution graph presented a total of 16,485 annotations with a mean level of 6.64 and standard deviation 2.58. A significant number of complete ORFs were found (5,34,791). The longest 5′ partial ORF (3354 nt) was annotated as protein SON-like isoform X2, whereas the longest 3′ partial ORF detected was the glutamate synthase [NADPH] large chain.

**TABLE 1:** Sequence statistics for LB and HB libraries.

Sample ID	No. of reads	Data in GB	GC%	Read length	% Q20	% Q30
Lib 1 LB84	33957484	5.128	42.0	151	97.980	86.070
Lib 2 LB88 + LB89	30187094	4.558	42.5	151	97.575	85.040
Lib 3 LB82 + LB87	35454238	5.354	38.5	151	97.660	84.785
Lib 4 HB133 + HB135	23770916	3.589	39.0	151	97.120	83.110
Lib 5 HB121 + HB122	26638640	4.022	41.5	151	97.435	84.700
Lib 6 HB104 + HB108	31325938	4.730	37.5	151	96.380	81.200

**TABLE 2:** Statistics of genome-guided transcriptome assembly completeness.

Attribute	Observation
Number of sequences	1,186,119
Longest sequence (nt)	16,755
Shortest sequence (nt)	201
GC-content (%)	36.17%
N50 sequence length (nt)	1,123
Core genes queried	429
Core genes detected	366
Complete	252 (58.74%)
Complete + Partial	366 (86.31%)
Scores in BUSCO format	C: 58% [D: 13%], F: 26%, and M: 14%
Average number of orthologs per core gene	1.27

### 3.2 Identification of differentially expressed transcripts, annotation, functional enrichment, and validation

**FIGURE 2:**
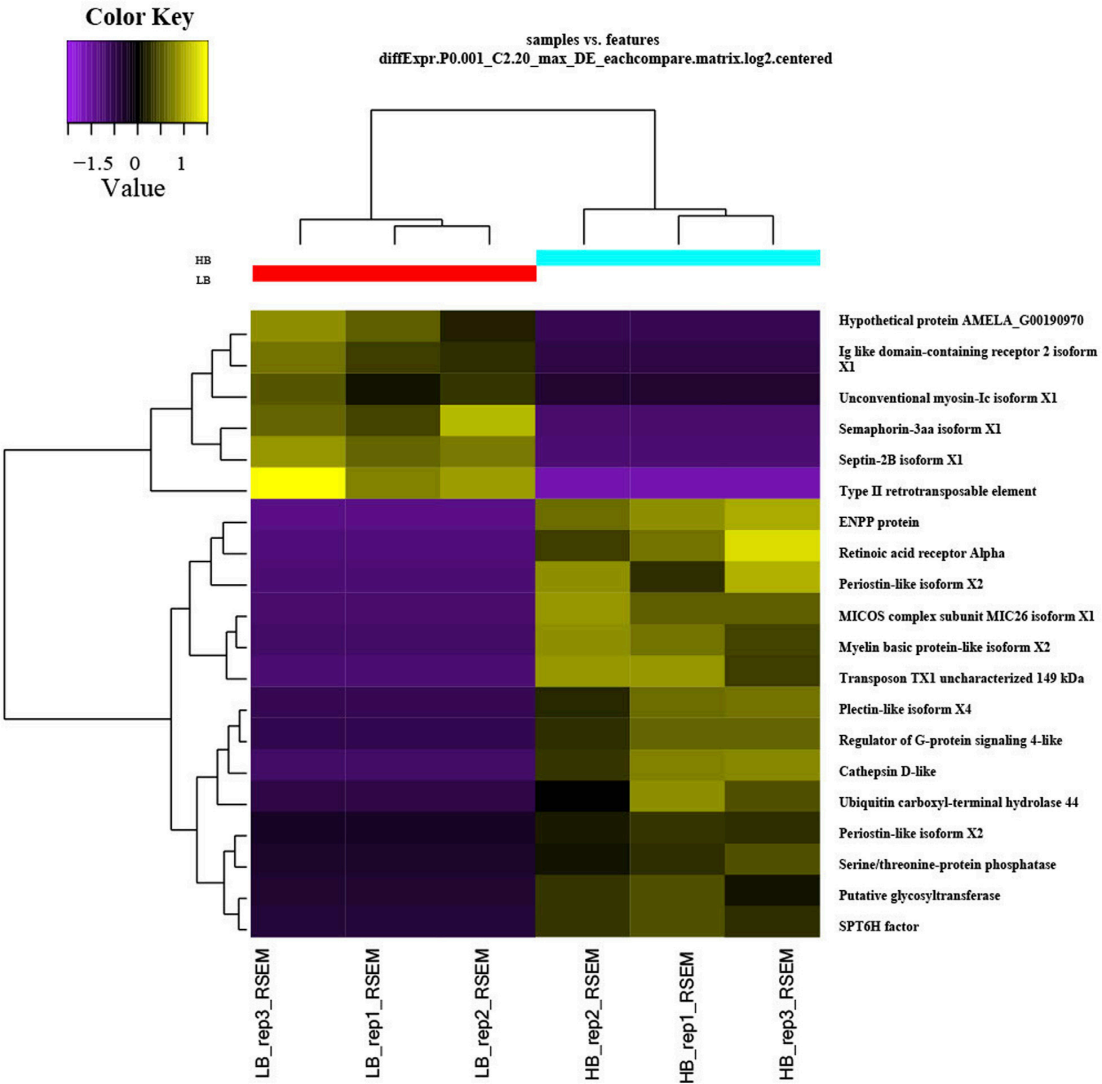
Heatmap of differentially expressed transcripts for LB and HB groups of *Labeo rohita*.

**FIGURE 3:**
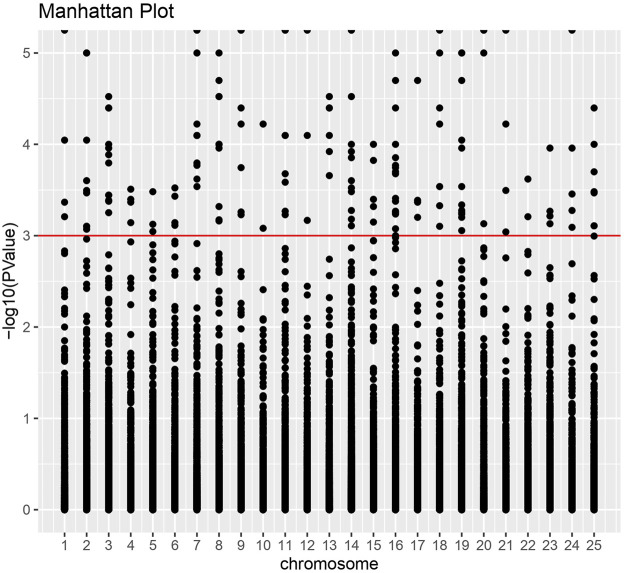
Manhattan plot for differentially expressed genes. X-axis indicates the number of chromosomes in the *Labeo rohita* genome, while the Y-axis shows-log10 (*p*-value) significance.

We discovered a significant proportion of transcripts that expressed differently between HB and LB groups on the two-fold change in expression with adjusted *p-value* < 0.5 × 10^−3^ and could obtain 451 upregulated and 181 downregulated transcripts. Heatmaps for DETs clearly categorized downregulation and upregulation of several genes in biological replicates of LB and HB groups (Figure 2). Functional annotation using GO categories revealed that “binding and cellular processes”-related GO terms were highly enriched (Supplementary Figure S2). Highly upregulated transcripts (more than 4-fold) were involved in biological processes such as muscle integrity, regeneration, epidermal differentiation, and regulation of growth factor binding. Downregulated transcripts were associated with cell cycle regulation, signal transduction, and ion channel binding. We could observe that DEGs were distributed across the chromosomes and a larger proportion was observed on chromosome no. 14, 16, and 19, indicating potential hotspots at respective locations (Figure 3). Functional annotation of DEGs indicated the most enriched enzyme class as “hydrolases,” followed by “transferases” and “oxidoreductases” (Supplementary Figure S3). KEGG pathways with ≥3 DEGs assigned and showing *p-value* < 0.05 were considered enriched. The KEGG pathway enrichment analysis of the DEGs indicated purine metabolism (Supplementary Figures S4, S10), thiamine metabolism, folate biosynthesis (Supplementary Figure S11), mTOR signaling fatty acid synthesis, and glycolysis/gluconeogenesis were highly enriched pathways in the HB group. Other than this, T cell receptor signaling and mucin-type O-glycan biosynthesis pathways were also enriched, indicating their potential role in enhanced immune capacity in the HB group. PPI network analysis revealed three major clusters, viz., eukaryotic translation initiation factors, structural proteins, and metabolic enzymes, along with growth factors. Among these, maximum protein interaction nodes were observed in eukaryotic translation initiation factors (Supplementary Figure S5).

A number of immune-related genes were highly upregulated in the HB group, a majority of them being immunoglobulins (Supplementary Table S3). Apolipoproteins belonging to L3 and L6 gene families, recently reported to have an active role in lipid metabolism in *Acipenser dabryanus* (Chen et al., 2021) in addition to immune-related functions, were found to be upregulated in the HB group. On the other hand, macrophages, lectins, and thrombospondin families were least represented (Supplementary Figure S6). Gene Ontology indicated major biological processes such as macromolecule catabolism (GO:0009057) and response to retinoic acid (GO:0071300). For category, “molecular processes,” calcium-dependent cysteine-type endopeptidase activity (GO:0004198), RNA polymerase II transcription factor activity, and sequence-specific transcription regulatory region DNA binding (GO:0001133) terms were enriched, while “cellular component” category reported enrichment of GO terms such as nuclear transcription factor complex (GO:0044798) and RNA polymerase II transcription factor complex (GO:0090575) (Supplementary Figures S7A–C). KEGG analysis showed involvement of the extracellular matrix (ECM) receptor interaction and phagosome followed by focal adhesion.

**FIGURE 4:**
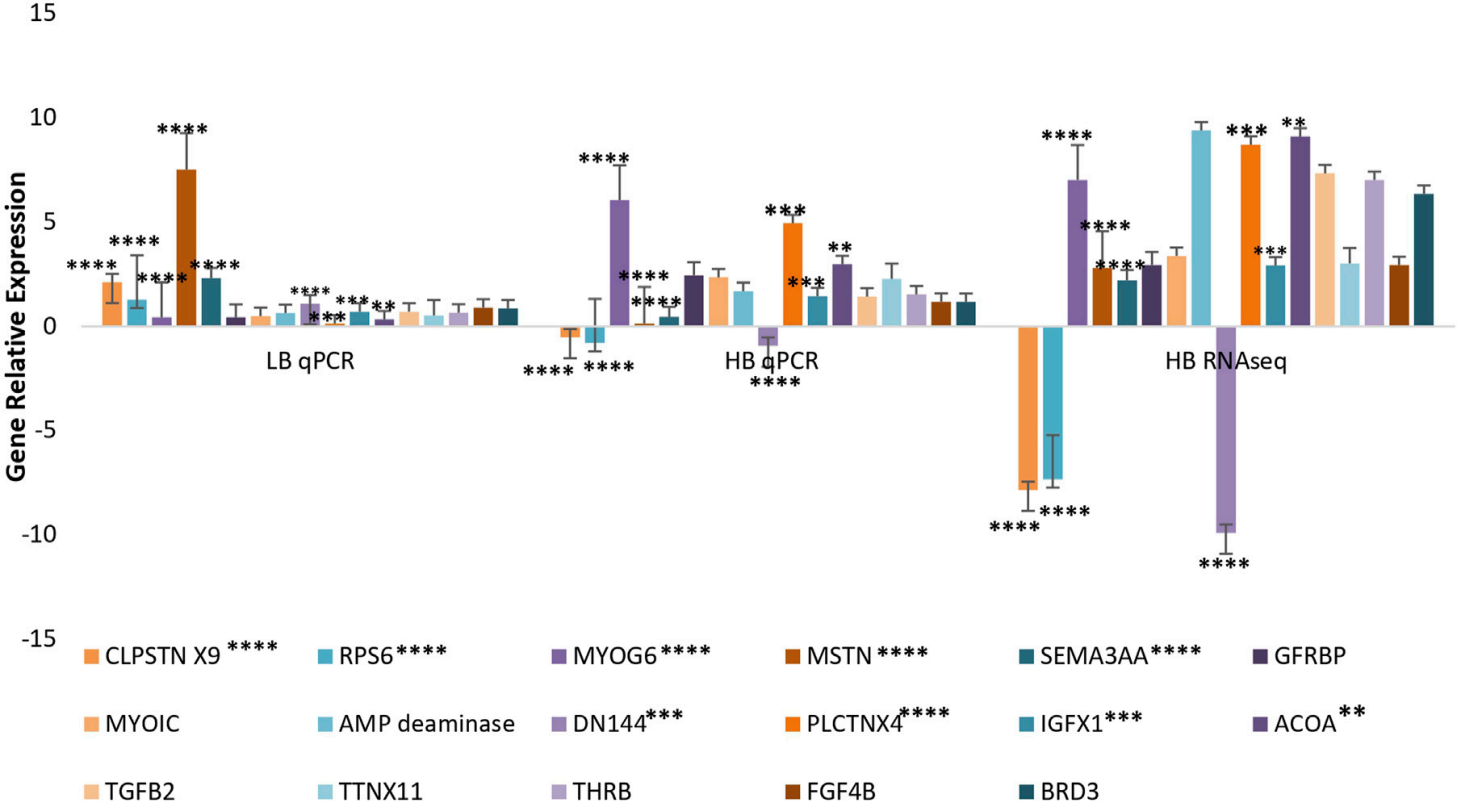
Validation of selected transcripts from RNA-seq data using relative gene expression in RNA-seq and qPCR data.

**FIGURE 5:**
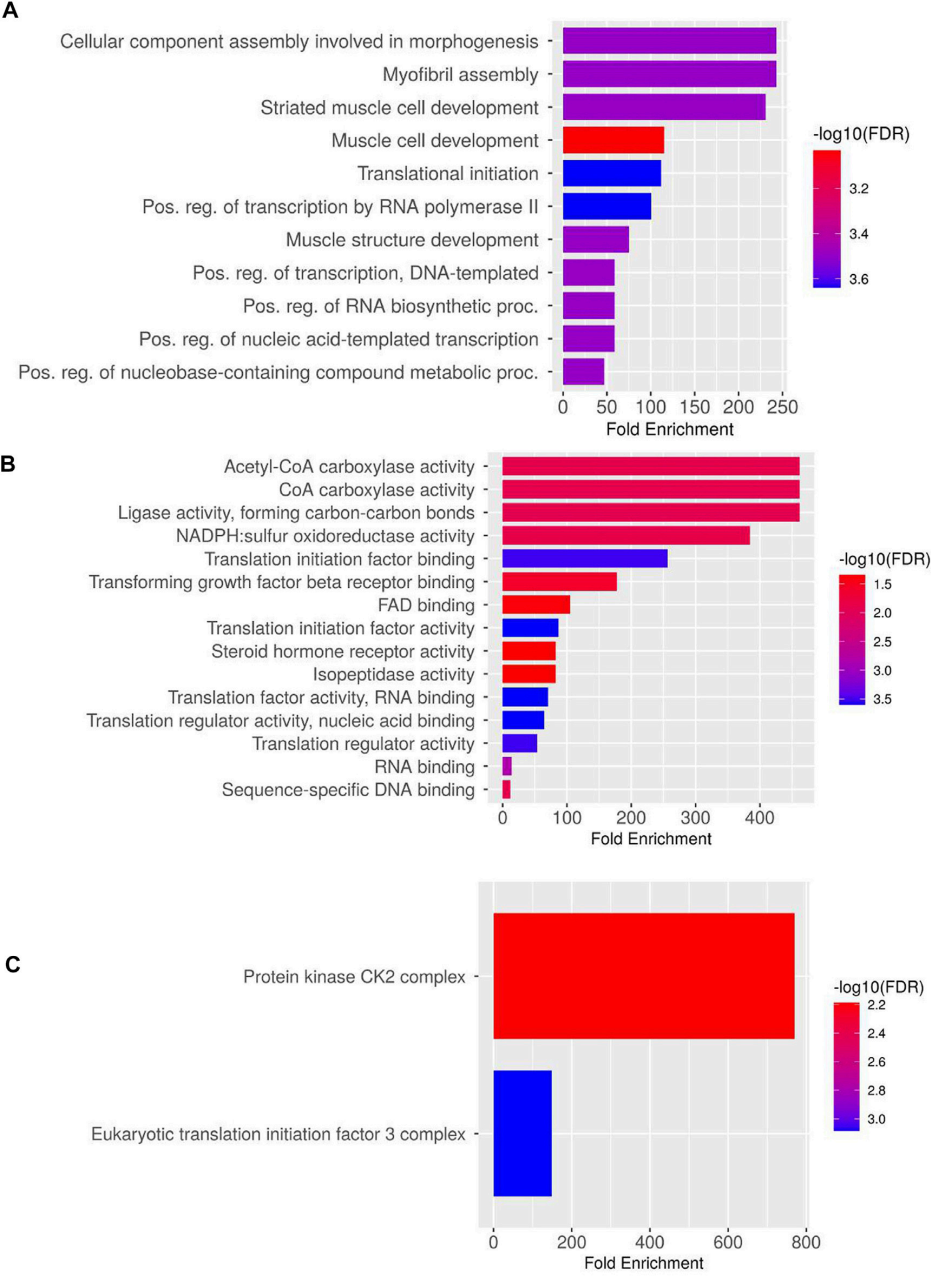
Gene Ontology and enriched KEGG pathways for validated transcripts: **(A)** biological processes, **(B)** molecular processes, and **(C)** cellular processes.

In total, 10 highly upregulated and 10 downregulated transcripts were selected for validation. Out of 20 transcripts tested for qPCR, 17 were successfully quantified in both HB and LB groups. Genes such as acetyl-CoA and transforming growth factor (TGF) showed variable upregulation in real-time and RNA-seq data, while variation was lesser for genes such as titin isoform X11, fibroblast growth factor 4B (FG4B), and growth factor receptor-bound protein 10-like isoform X1 (GRBP), indicating complementarity of RNA-seq and qRT data (Figure 4). Correlation and *t*-test employed for comparing overall expression data of RNA-seq and qPCR revealed positive correlation (0.51) and *p-value* > 0.05 (0.139) which was insignificant, and hence it was concluded that no significant difference between qPCR and RNA-seq data was observed (Supplementary Figure S8). However, at transcript level, comparison between qPCR LB, HB, and RNA-seq HB data indicated significant variation between expression levels of calpastatin, sema3AA, and RPS6 primarily because of their upregulation in the LB group and contrast downregulation in the HB group. We could also observe a sharp upregulation in the myostatin expression LB group and considerable downregulation in the HB group (Figure 4). Figure 4 shows that the expression of AMP deaminase and myogenic 6 is quite significant (*p-value* < 0.005) as noted by asterisks in the HB group and insignificant in other groups. In addition to this, the paired *t*-test for HB and LB groups indicated *p-value* < 0.05 (0.042), depicting significant difference of gene expression between both groups. PPI network analysis revealed 38 nodes with 51 edges, and average local clustering coefficient was 0.63. The GO and KEGG analysis for qPCR validated transcripts revealed the most significant GO terms such as “morphogenesis,” “myofibril assembly,” “striated muscle development,” and “muscle cell development” under the biological processes category (Figure 5A). Terms such as acetyl-CoA carboxylase activity, ligase activity, NAPDH: sulfur oxidoreductase activity, translation initiation factor binding, TGF beta receptor binding, and FAD binding were mostly enriched under category “molecular processes” (Figure 5B). The Cellular component category had two terms enriched, viz., protein kinase CK2 complex and eukaryotic translation initiation factor 3 complex (Figure 5C).

### 3.3 Transcription factor enrichment analysis and prediction of miRNA target interaction

**FIGURE 6:**
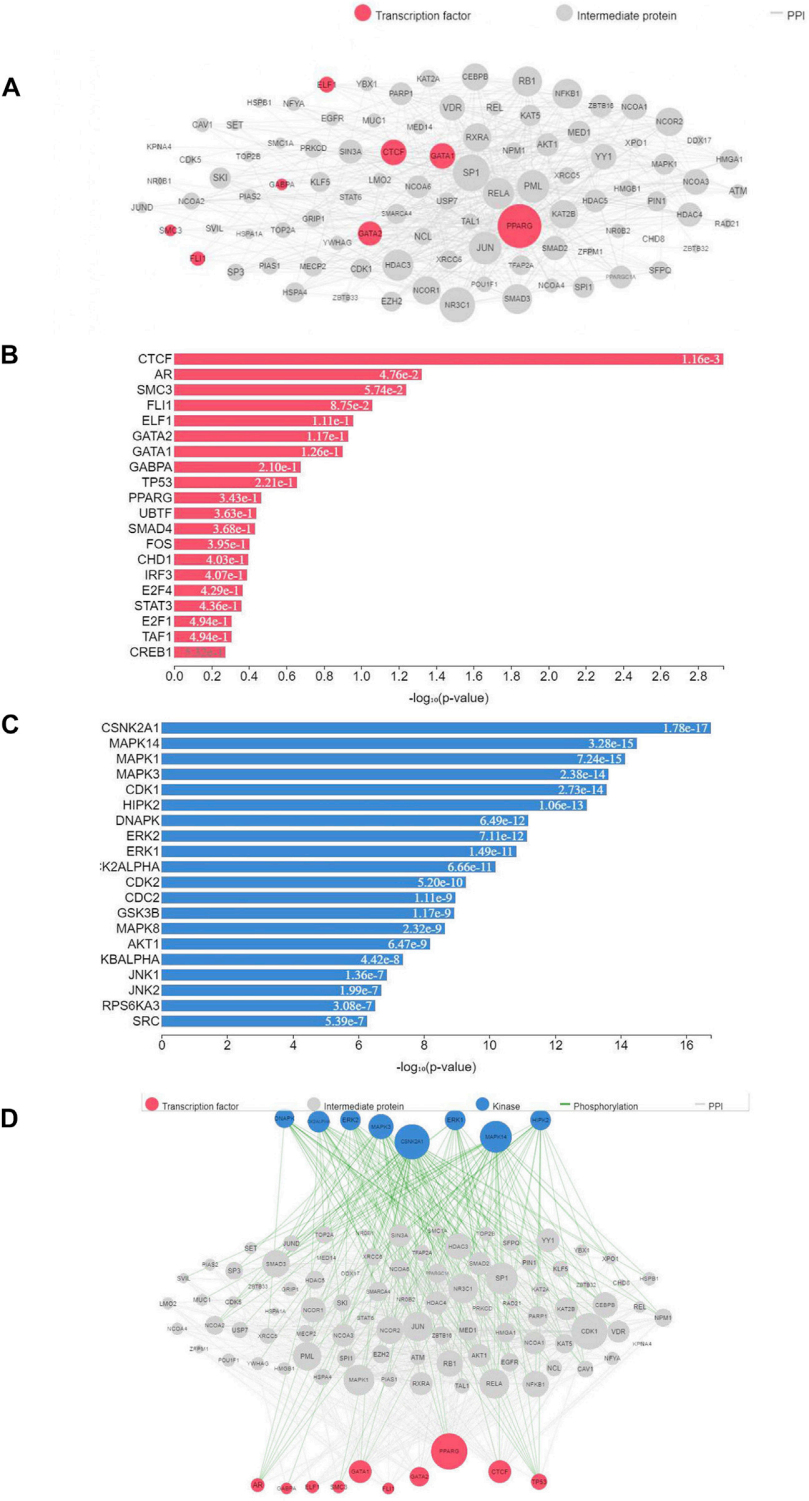
Transcription factors associated with DETs and their interactions with related proteins and kinases. **(A)** TFs associated with DETs; **(B)** PPI interactions of enriched TFs; **(C)** kinase enrichment analysis of transcription factors; **(D)** prediction of the upstream regulatory network of DETs using the eXpression2Kinases network. TFs are shown in red, while kinases are depicted in blue; size of the bubble indicates the significance of TFs and kinases. Gray dots represent intermediate proteins, while white lines indicate interactions.

TFEA was performed on 39 top predicted transcription factors attained from significant DETs obtained as a result of differential expression analysis. Top predicted TFs included CTCF, AR, SMC3, FLI1, ELF1, and PPARG. (Figure 6B). These TFs are predicted to interact with POU1F1, EGFR, SIN3A, MUC1, SP3, LMO2, STAT6, RXRA, RELA, FAB2, and SMAD2 (Figure 6A). Furthermore, the prediction of likely regulators of PPIs, protein kinases (PKs), was performed using kinase enrichment analysis (KEA) with gene set libraries from kinase-substrate interaction databases. The top significant PKs were CSNK2A1, MAPK1–14, CDK1, and HIPK2 (*p-value* < 0.01) (Figure 6C). In addition to this, the eXpression2Kinases (X2K) network was created for prediction of an upstream regulatory network of DETs inferred from TFEA (Figure 6D). A total of 26 miRNA target interactions (*p-value* < 0.05) were found to be associated with significant DETs. Most of them were found to have positive or negative regulation of cell proliferation and apoptosis and were associated with genes such as thyroid receptor, acetyl-CoA, and EO GT1R (Table 3). Top five enriched MTIs associated with transcription factors were miR-17-5p, miR-16-5p, miR-24-3, miR-20-5p, and miR-21-5p (Supplementary Figure S9).

**TABLE 3:** Identification of microRNA targets associated with differentially expressed genes.

MTI Term	P-value	Odds Ratio	Combined Score	Associated Genes	Function	Reference
miR-425-5p	0.0047	9.580	51.348	THRB;EOGT;EIF3C	Cell proliferation	Wu et al. (2021)
miR-597-5p	0.0059	18.823	96.538	ACACA;IGF1R	Inhibition of cell proliferation and migration	He et al. (2017)
miR-592	0.007	17.009	84.022	ACACA;IGF1R	Cell proliferation	Pan et al. (2021)
miR-3690	0.009	14.493	67.280	ACACA;HBEGF	Cell cycle progression	Shen et al. (2020)
miR-15b-3p	0.010	13.811	62.883	THRB;IGF1R	cell proliferation and migration	Wei et al. (2020)
miR-5002-5p	0.012	12.624	55.375	THRB;ACACA	Cell proliferation	Berillo et al. (2013)
miR-4254	0.013	11.939	51.142	ACACA;HBEGF	Tissue repair and regeneration	Dao et al. (2018)
miR-4433b-5p	0.015	11.324	47.408	ACACA;IGF1R	Cell to cell communication	Carvalho et al. (2022)
miR-4261	0.016	10.770	44.092	EOGT;ACACA	Cell proliferation	Jiao et al. (2017)
miR-194-5p	0.020	9.7006	37.857	IGF1R;HBEGF	Regulator of Apoptosis; Target gene is IGF1R	Niu et al. (2021)
miR-4520-5p	0.020	54.198	209.49	EOGT	Cell proliferation, Stress biomarker	Rong & Liu (2020)
miR-590-5p	0.020	54.198	209.49	TNS1	Negative regulation of NF90/VEGFA signalling axis	Q. Zhou et al., (2016)
miR-4636	0.023	48.173	181.187	ACACA	Inhibition of tumor cell progression	Tang et al. (2020)
miR-320c	0.026	8.321	30.158	THRB;EIF3C	Apoptosis regulator	Lim et al. (2020)
miR-296-3p	0.027	39.411	141.134	IGF1R	Cell proliferation	Zhou et al. (2020)
miR-6857-3p	0.028	8.0913	28.914	BRD2;ACACA	NA	
miR-3161	0.029	7.8033	27.376	THRB;EOGT	NA	
miR-344b-3p	0.032	33.344	114.345	THRB	Negative regulator of TLR2 signalling pathway	Xu et al. (2017)
miR-6084	0.032	33.344	114.345	IGF1R		
miR-6727-5p	0.041	25.493	81.132	IGF1R	Cell proliferation	Liu et al. (2017)
miR-494-3p	0.041	6.476	20.568	ACACA;IGF1R	Mitochondrial biogenesis	Lemecha et al. (2018)
miR-465b-5p	0.043	24.076	75.347	THRB	Negative regulator of ESC differentiation	Sun et al. (2019)
miR-465a-5p	0.043	24.076	75.347	THRB	Critical suppressor of osteoblastogenesis	Zheng et al. (2021)
miR-465c-5p	0.043	24.076	75.347	THRB		
miR-1237-3p	0.045	6.200	19.218	TNS1;HBEGF	Associated with reduced chordoma invasion	Zou et al. (2015)
miR-466f-5p	0.048	21.666	65.687	EOGT	Cell differentiation	Gong et al. (2014)

### 3.4 Coding SNP identification and annotation

A total of 39,452 cSNPs were discovered and after stringent filtering, and around 39,158 high-quality cSNPs were identified with 38,044 transitions (Ts) and 30,800 transversions (Tv) with the Ts/Tv ratio of 1.23. A total of 5,816 A/G and 6,093 C/T base changes were observed (Table 4). More cSNPs were present in the downstream region (20.18%) than those present upstream (19.2%) (Table 5). A total of 4,589 cSNPs (57.48%) were missense and might have effects on protein expression. On the other hand, 40% of the cSNPs (3,270) were silent effectors. Approximately 124 cSNPs were classified as nonsense cSNPs. In total, 95% of the cSNPs had a modifier effect on protein expression. Our studies revealed 12 cSNPs associated with significant validated DEGs such as myogenic factor 6, fibroblast growth factor, growth factor receptor-bound protein 10-like, IGF-1-like, and TGF β (Table 6). Upon comparing the chromosomal location of these SNPs, we could detect more than one cSNP closely spaced in chromosome no. 4, 14, and 19 (Table 6). The highest number of SNPs was present on chromosome no. 14. These genes could be candidates for QTL search, and hence further studies are required to rightly pinpoint the QTLs and elucidate their association with growth in improved rohu.

**TABLE 4:** Base changes attributed to cSNPs.

	A	C	G	T
**A**	0	2166	5816	3283
**C**	2047	0	1354	6093
**G**	5201	1154	0	2304
**T**	3162	4566	2012	0

**TABLE 5:** cSNP annotation and number of effects by type and region.

Type of variant (SNP)	Count	Percent
Downstream gene variant	44,882	20.18
Intergenic region	13,896	6.28
Intron variant	27,219	12.21
Missense variant	4,523	2.03
Non-coding transcript exon variant	2,429	1.09
Splice acceptor variant	81	0.04
Splice donor variant	135	0.06
Splice region variant	398	0.18
Start lost	1	0.00
Stop gained	124	0.06
Stop lost	65	0.03
Stop retained variant	21	0.01
Synonymous variant	5,638	2.53
Upstream gene variant	42,719	19.16

Region of variant (SNP)	Count	Percent
Downstream	44,882	20.18
Exon	12,718	5.72
Intergenic	13,896	6.25
Intron	26,799	12.05
Splice site acceptor	81	0.04
Splice site donor	131	0.06
Splice site region	336	0.15
Transcript	59,102	26.58
Upstream	42,719	19.21

**TABLE 6:** cSNPs associated with differentially expressed genes and their effects.

Gene name	Function	Accession	Chromosome	Start	Stop	Length	cSNP	Position	SNP effect
Retinoid acid receptor alpha-B isoform X1	Cellular growth and differentiation	NC_066871.1	3	17723931	17808700	453	G/T	17796406	Intron variant and modifier, high
Myogenic factor 6	Muscle differentiation	NC_066872.1	4	27560948	27562329	239	G/C	27601011	Missense variant, moderate
Insulin-like growth factor I isoform X1	Growth hormone regulation	NC_066872.1	4	32034024	32045647	149	A/T	32017624	Upstream gene variant and modifier, high
Transforming growth factor beta-2 proprotein	Regulator of cell proliferation	NC_066882.1	14	22043194	22055303	414	A/G	22069302	3′ UTR variant and modifier, high
Meprin A subunit beta-like isoform X1	Cytokine processing and inflammation	NC_066882.1	14	32556739	32577283	646	A/T	32571335	Protein coding, high
Eukaryotic translation initiation factor 4E-binding protein 3-like	Translation initiation	NC_066882.1	14	21136243	21141167	112	C/G	21136224	3′ UTR variant modifier, moderate
							T/A	21141033	Splice donor variant and intron variant, high
Growth factor receptor-bound protein 10 isoform X1	Regulator of the Wnt pathway	NC_066884.1	16	29492597	29558237	600	T/C	29581464	Upstream gene variant modifier, Moderate
Titin isoform X11	Muscle elasticity	NC_066885.1	17	9353801	9392343	3547	T/C	9379989	Splice donor variant and intron variant, high
							C/T	9380005	Missense variant, moderate
Thyroid hormone receptor beta isoform X1	Muscle development, metabolism	NC_066887.1	19	319012	347736	415	C/T	338329	Intronic modifier, high
Casein kinase II subunit beta	Cell metabolism and differentiation	NC_066887.1	19	7748857	7753726	215	A/G	7748458	3′ UTR modifier, high

## 4 Discussion

The present study aims to identify genes associated with the HB group of improved rohu of the tenth generation selected for faster growth, which will be helpful for identifying faster growing candidates for breeding. Muscle growth in mammals occurs primarily through hypertrophy, with little cellular proliferation. In contrast to mammals, fish muscle growth is accomplished through both hypertrophy and hyperplasia, particularly in large bodied and fast-growing fish, while slow-growing and small-sized fish primarily grow because of hypertrophy and display a slower recruitment rate for muscle fibers (Liu et al., 2020). Another distinguishing characteristic of muscle growth in fish compared to other vertebrates is that it is continuous. This fact explains why, after sexual maturation, fish continues to grow with significant rates of muscle hyperplasia, in contrast to many mammals (Vélez et al., 2017).

### 4.1 Sampling, mRNA sequencing, QC, and assembly

For sampling, we selected individuals on the basis of their estimated breeding values (EBVs). The EBV is the genetic superiority of individuals contributing to the next generation. The higher the EBV, the better is the individual’s ability to contribute selected alleles for a particular trait to the next generation. As we had to identify genes linked to high body weight concurrent with high EBV, we selected six fish/groups, i.e., LB and HB, based on earlier reports (Danzmann et al., 2016; Lin et al., 2019; Shen et al., 2022). Clean and trimmed reads generated had GC content ranging from 37% to 42%, which is similar to previous reports (Das et al., 2020; Jaiswal et al., 2021). BUSCO analysis indicated that our transcriptome had partial to complete gene information for 86.31% of the genes when compared with the eukaryote orthologous set. CD-HIT EST revealed 12,97,979 UniGene clusters, which upon annotation were found to be involved in several metabolic and signaling pathways. The longest 5’ prime partial ORF (3354 nt) annotated as protein SON-like isoform X2 was significantly upregulated (4-fold) in the HB group. Majorly known in humans, SON protein is a huge serine-/arginine-related protein which has an active role as a splicing cofactor that promotes effective splicing during cell cycle progression (Ahn et al., 2011). The longest 3′ partial ORF, glutamate synthase [NADPH] large chain, is mainly involved in glutamate, nitrogen metabolism, cell integrity, and protein synthesis and is documented to have a role in muscle growth (Rufino-Palomares et al., 2016).

### 4.2 Identification of differentially expressed transcripts, annotation, and functional enrichment

For this particular study, we had taken six HB and five LB adult rohu samples of the same age group (2 years) and one spawning batch belonging to the tenth generation of the ongoing selective breeding program, raised in a common environment, i.e., pond. The HB group contained superior genetic makeup of rohu being selected for more than 10 generations for higher growth rate. The LB group represented rohu having low breeding value and exhibited lesser growth as compared to the HB group. Differential gene expression revealed 451 up and 181 downregulated genes between HB and LB group. Selective breeding, which is going on continuously for 12 generations, may presumably have favorable alleles in their respective lines and might have resulted in a large number of DEGs. Historically, Indian major carps including rohu, *Labeo rohita* originally belonged to the Ganga river system and its tributaries. In river, they naturally breed in monsoon season in the presence of environmental conditions such as low temperature, rainy cloudy weather, and moving water system. In stagnant/confined water bodies such as ponds, however, they mature, but do not breed/spawn there unless they are artificially induced using commercially available GnRH analogs such as ovaprim/ovatide or pituitaries (Kaui and Rishi, 1986; Bais, 2018), mostly practiced in specialized carp hatchery systems. Hence, HB and LB groups, though raised together, do not breed between or within groups unless they are artificially induced. Furthermore, continuous selective breeding of higher breeding value individuals led to increased frequency of alleles favorable for higher body growth might have very well influenced the levels of transcript expression in major growth, metabolism, and immunity-related pathways in the HB group as evident from our results. Though we have taken equal numbers of males and females (three males and three females) in each sampling group, its effect on a large number of differentially expressed genes cannot be ruled out. It is noteworthy that most of the researchers have used both males and females in their studies of differentially expressed genes related to body growth (Lin et al., 2019; Guan, Qiu, and Feng-Liu, 2020; Shen et al., 2022). In this study, we could find that upregulated transcripts were specific to major categories such as cellular proliferation, fatty acid metabolism, and regulation of growth factor binding, which contributes to the elevated hypertrophic conditions favoring more body growth. Apart from this, many genes such as titin, actin, myosin, and myotrophin which are associated with muscle integrity, hypertrophy, transcription, ribonucleoprotein packaging and transport, and chromatin remodeling (Alves-Costa et al., 2015) were upregulated. Furthermore, the purine and thiamine metabolism pathways followed by the folate biosynthesis pathway were most significantly enriched and are reported to improve growth performance (Jamalzad Falah et al., 2020) (Supplementary Figure S4) (Supplementary Figures S10, S11).

#### 4.2.1 GH–IGF system, PI3K/AKT, and mTOR pathways hint elevated protein synthesis

The GH–IGF system is primarily responsible for vertebrate growth and development. IGF1 is one of the most studied growth factors which influence muscle growth. Growth hormone (GH) is the activator for the Janus kinase (JAK)-signal transducer and activator of transcription (STAT) signaling pathway (Bergan-Roller & Sheridan, 2018). IGF1 has been linked to growth, metabolism (Castillo et al., 2004), development (Moon & Choi, 2020), reproduction (Reinecke, 2010), and osmoregulation (Chandhini et al., 2021) in fish and shellfish. The IGF-binding proteins promote IGF binding to the IGF receptor. A greater abundance of IGFR1 in fish muscle than that in insulin receptors indicates that IGFR1 is more important in the regulation of muscle function than IR in fish (Liu et al., 2020). In this study, we could find elevated expression (>3 FC) of IGF-related genes such as insulin-like growth factor I isoform X1, insulinase family protein, and insulin receptor-like proteins. Significant upregulated eIF-3 subunit C isoform X1 (7-fold) (Supplementary Table S3) displayed numerous protein interactions with other eukaryotic translation initiation factors, such as eIF3d, eIF3g, eIF3f, and eIF4e1c (Supplementary Figure S5. The eIF-3 complex precisely targets and inducts the translation of mRNAs involved in cell proliferation, and hence its upregulation indicated a stimulative effect on protein synthesis in the HB group as compared to the LB group. Apart from this, reports of a contributory effect of IGF1 on glucose uptake other than cellular proliferation have also been reported (Montserrat et al., 2012). Many signaling pathways are involved in the regulation of muscle mass. The phosphatidylinositol 3-kinase/PI3K/AKT pathway, which activates protein synthesis and inhibits protein degradation, is a key player in muscle mass regulation. mTOR is a ser/thr kinase that detects intracellular and environmental changes and synchronizes a variety of cellular processes such as cell growth, autophagy, and is a master regulator in controlling skeletal muscle mass (Yoon, 2017). MTOR regulates the phosphorylation of key regulators of mRNA translation and ribosome synthesis, and mTORC1 promotes protein synthesis. In this study, we could reveal the upregulation of RIO2, WNK4, WNK4-like, and SMG1-like serine/threonine protein kinases in the HB group which are involved in PI3K/AKT pathways. In response to hormones, nutrients, growth-associated factors, and signals associated with stress, they are the central regulators of cell metabolism and growth (Saxton & Sabatini, 2017).

#### 4.2.2 Efficient ubiquitin–proteasome system improved muscle regeneration

A major intracellular protein degradation system, the ubiquitin–proteasome system (UPS), is important for muscle homeostasis and health. Myoblast cells are produced from stimulated satellite cells and then go through the series of phases to become myofibers, including proliferation, differentiation, fusion, and maturation (Chargé & Rudnicki, 2004; Tajbakhsh, 2009). Recently, better growth performance in common carp was found to be associated with elevated UPS activity concurrent with the activation of protein synthesis (Sun et al., 2018). Other studies have found that the enhanced UPS activity is critical in satellite cell (muscle stem cells) proliferation and is associated with augmented muscle regeneration (Kitajima et al., 2018; Kitajima et al., 2020). In this study, we could find that several genes associated with the UPS system such as E3 ubiquitin-protein ligase SMURF2-like, E3 ubiquitin-protein ligase TTC3-like, E3 ubiquitin-protein ligase MYCBP2-like, and E3 ubiquitin-protein ligase XIAP-like isoform X1 were highly upregulated in the HB group, pointing toward the active development of satellite cells required for cell proliferation. Ubiquitin carboxyl-terminal hydrolase L1 (UCHL1 or PGP9.5) positively regulates myoblast proliferation (Gao et al., 2017). This finding was similar to our results where we could observe high upregulation (up to 7-fold) of UCH isoforms such as ubiquitin carboxyl-terminal hydrolase 15 isoform X3, UCH 2-like isoform X2, UCH MINDY-3, and UCH 4, which might be positively involved in myoblast proliferation in the HB group and hence beneficial for muscle regeneration and repair.

#### 4.2.3 Immunity-related genes contributed to muscle growth

As per earlier reports in improved rohu, disease resistance to aeromoniasis is positively correlated with the growth rate (estimated genetic correlation: 0.43) (Mahapatra et al., 2017). We could detect substantial upregulation of polymeric immunoglobulin receptor (pIgR)-like gene which plays an active role in mucosal surface defense by modulating transport of polymeric Ig (Turula & Wobus, 2018). Recently, its role in tumor cell growth and proliferation has also been unveiled (Dewdney & Hebbard, 2018), hinting its indirect effect in regulating muscle growth besides imparting immunity. In addition to this, upregulation of HSP20, HSP70, and HSP90 chaperones indicated marked protein folding efficiency for newly synthesized proteins, refolding of misfolded ones, and regulation of protein activity (Mayer and Bukau, 2005), suggesting prominent protein synthesis in the HB group. Furthermore, upregulation of mucin isoform indicated an active role in imparting innate immunity and modulation of mucus secretion (Marcos-López et al., 2018), thereby imparting better disease resistance. Functional annotation of immunity-related genes indicated their role in biological processes such as “macromolecule catabolic process” followed by “cellular response to retinoic acid” (Supplementary Figure S7A). Apart from hinting at elevated catabolic processes, high expression of retinoic acid receptor alpha indicates significant levels of retinoids, which are essential for proper innate and adaptive immune responses. Moreover, its role in regulating cellular differentiation is also well-known (Chen and Catharine Ross, 2004; Cunningham and Gregg, 2015) According to Chen and Catharine Ross (2004), retinoic acid may enhance immunity by inducing differentiation of myeloid and lymphoid cells, but full elucidation of mechanisms of its activity in the immune system remains unknown. Calpastatin expression is suppressed (−7 FC) in the HB group, which hints at elevated levels of calcium-dependent cysteine proteases, popularly known as calpain. Under the molecular category, “calcium-dependent cysteine-type endopeptidase activity” was the highest enriched GO term complementing elevated calpain levels which are responsible for cellular inflammatory responses (Supplementary Figure S7B). Enrichment of GO term “nuclear transcription factor complex” under the cellular compartmentalization category (Supplementary Figure S7C) hints at the significantly active NF-κB signaling pathway which regulates inflammation, cell proliferation, and immunity responses. Siglec-6 is also known as obesity-binding protein 1 (OB-BP1) and CD antigen. CD327 is mainly involved in mediating sialic acid-dependent binding to cells, and through glycan recognition, siglec regulates the functions of cells in the innate and adaptive immune system (Crocker et al., 2007). Due to their upregulation in the HB group, these proteins could have additional roles in growth, which may be studied in future.

Furthermore, we validated 17 transcripts out of 20 which showed a similar trend with RNA-seq data, but the overall expression average was on the lower side. Validated transcripts included thyroid hormone receptor beta, acetyl-CoA, and fibroblast growth factor 4B responsible for cellular proliferation and cell cycle progression and muscle building, growth, and regeneration (Edwards et al., 2009; Shi & Tu, 2015; Shibata et al., 2016). In addition to this, upregulated expression of IGF1-like in the HB group hinted its role in muscle growth by building new satellite cells, giving rise to new myofibrils and stimulating the mTOR pathway. Other than this, many structural genes such as plectin and myogenin were found to be upregulated, indicating their role in muscle growth. Plectin is essential for C2C12 myoblast differentiation and proliferation and for regulating the expression of atrophy-related genes (atrogin-1 and muRF-1) (Yin et al., 2021). Furthermore, upregulation of unconventional myosin-Ic-like isoform X1 (myoIc) known to improve microfilament motor activities like motility and vehicle transport (Fili & Toseland, 2019) was observed in the HB group. Upregulation of thyroid hormone receptor beta indicates involvement in increasing GH, adrenocorticotropic hormone (ACTH), and alpha-melanocyte stimulating hormone (alpha-MSH) which acts by binding to thyroid receptors (Kongchum et al., 2010). Furthermore, TH receptors are known to regulate the rate of transcription by modulation of T3 binding and are associated with the retinoid x receptor (RXR) along with regulation of mitogen-activated protein kinase (MAPK) or phosphatidylinositol 3-kinase (PI3K) pathways (Hiroi et al., 2006).

### 4.3 Gene ontology analysis of validated DETs suggests the significant association of growth with cellular proliferation and fatty acid metabolism

The GO analysis indicated the significant involvement of DETs in morphogenesis, striated muscle development, and translation initiation under biological processes, which might be due to continuous body growth of fish throughout its lifespan. Enrichment of GO terms such as muscle structure development and post transcriptional regulation of transcription by RNA Pol II was also evident. Regulation of transcription by RNA Pol II is further influenced by changes in chromatin structure, interactions of regulatory elements, promoters, co-regulators, and mechanisms associated with progression of transcription (Linzer et al., 2021). Striated muscle cell development was also flagged as an important biological process in this study, which hinted at cellular machinery working toward development of myofibrils, an important muscle fiber component for inducing muscle hypertrophy. Among molecular processes, acetyl-CoA carboxylase (ACC) activity was the most enriched term. ACCs have a central role in fatty acid metabolism and insulin signaling pathway (Supplementary Figure S12). It catalyzes the carboxylation of acetyl-CoA by ATP to form malonyl-CoA (Harwood Jr, 2005), which is a critical metabolic signal for controlling fatty acid production and utilization in response to environmental changes. Significant upregulation of ACC (9-fold) and mitochondrial malonyl-CoA decarboxylase (6.9-fold) in the HB group hints at enhanced fatty acid biogenesis attributed to increased body weight (Sakamoto et al., 2000). In our study, KEGG analysis showed a similar trend as in enriched molecular and biological processes such as MAPK and FoxO signaling pathways along with major metabolic pathways including fatty acid biosynthesis, adherens junction, TGF, and insulin signaling pathway. MAPK cascades have been shown to play an important role in the conversion of extracellular signals to cellular responses. There are at least three MAPK families known: extracellular signal-regulated kinase (ERK), Jun kinase (JNK/SAPK), and p38 MAPK, which are known to be associated with many complex cellular processes, including proliferation, differentiation, protein synthesis, development, transformation, and apoptosis (Zhang & Liu, 2002). Usually located downstream of many growth factor receptors, the MAPK pathway indicated upregulation of receptor tyrosine kinase (RTK) in our study (Supplementary Figure S13), which are cell-surface transmembrane proteins that function as signal transducers. They control vital cellular processes such as proliferation, metabolism, and apoptosis (Dev et al., 2021). Along with upregulation of RTKs, TGFB, TGFB receptors, and MEF2C, which are a part of JNK and p38 MAPK pathway, also had elevated profiles and hinted at their involvement in cell proliferation (Pon & Marra, 2016).

### 4.4 Transcription factor enrichment analysis and miRNA target identification

**FIGURE 7:**
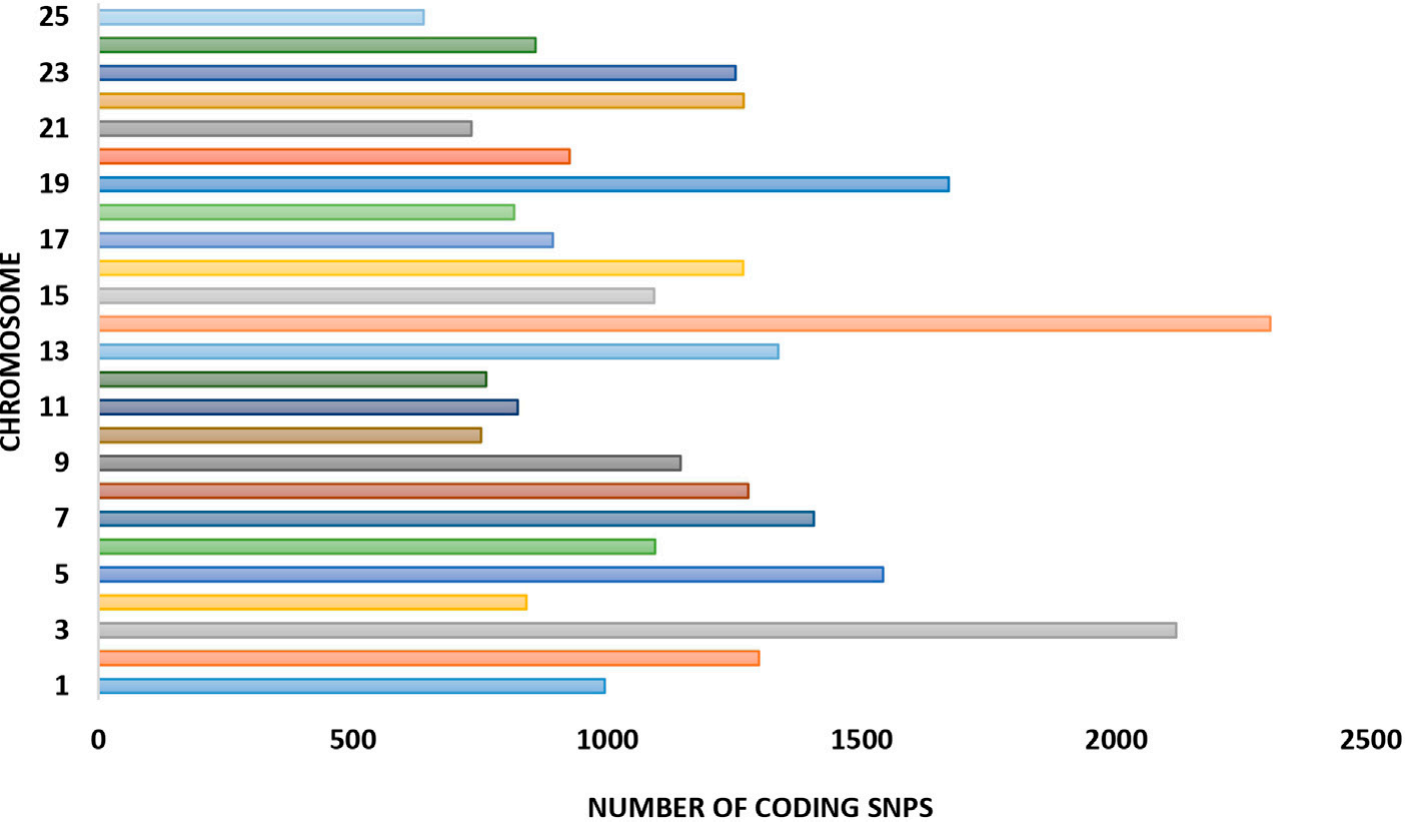
Distribution of cSNPs across *Labeo rohita* chromosomes.

TFs play a vital role in transcription initiation by binding to targeted sequences of DNA, thereby regulating RNA polymerase activity. Understanding transcriptional regulation helps track the dynamics of gene expression in response to genetic or environmental variations (Pascual-Ahuir et al., 2020). There are limited reports related to information on TFs playing a key role in growth regulation such as POU1F1 (POU class 1 homeobox 1) (Wang et al., 2017), homeostasis, and stress regulation (Sampieri et al., 2019). Sahoo and colleagues discovered 13 classes of TFs from liver and muscle tissues of rohu, viz., bZIP, bHLH, and CSD (Sahoo et al., 2022). Similar observation was also made in our results (Figure 6A) and in addition to it, we could find that LRRFIP followed by the bHLH and leucine zipper class (bZIP) was highly represented in the rohu muscle. On the other hand, TFEA for significant DETs indicated their involvement pathways associated with cell growth and chromosome architecture. The PPI network analysis of peroxisome proliferator-activated receptor (PPARG), a metabolic nuclear receptor involved in energy homeostasis and cell proliferation (Hernandez-Quiles et al., 2021), presented its interaction with SMAD2, RELA, PML, KAT2B, and JUN (Figure 7B), out of which many are associated with biological functions such as cellular proliferation, immunity, and apoptosis (Ungefroren et al., 2011; Lee, 2017; Venezia et al., 2021).

A transcriptional repressor and insulator, CTCF, highest enriched TF in our study, mediates chromatin looping and boundary establishment with chromatin structure (Fang et al., 2020). According to Stik et al. (2020), CTCF is needed for the expression of genes specific to lineage during cell differentiation. As it is highly enriched in our TF enrichment data, it can be concluded that its role in cell differentiation is not only limited to juvenile stages of life but also in adult stages as well. PPI studies revealed the interaction of CTCF with SIN3A, GATA1, GATA2, and NCOA6. A pleiotropic coregulator, NCoA6 plays a role in growth, development, wound healing, and energy homeostasis maintenance. NCOA6 acts as a coregulator for estradiol (E2)/ERα–activated GREB1 transcription intended for regulation of cell proliferation (Mahajan & Samuels, 2008; Tong et al., 2019). The eXpression2Kinases (X2K) network created for prediction of the upstream regulatory network of DETs inferred from TFEA indicated the involvement of major PPI regulators, CSNK2A1 and MAPK14. Both of the protein kinases are known to be associated with cellular growth progression, transcription, and apoptosis. The MAP kinase signaling pathway member, MAPK14, plays an imperative role in cellular responses associated with environmental stimuli such as physical stress or proinflammatory cytokines causing transcription factor activation (Notch et al., 2012). The top five enriched miRNA targets associated with transcription factor were miR-17-5p, miR-16-5p, miR-24-3p, miR-20-5p, and miR-21-5p, which were mostly associated with cell proliferation (Stoen et al., 2021; Yan et al., 2019), cell cycle regulation (Wang et al., 2021), and apoptosis modulation (Xiao et al., 2018).

### 4.5 cSNP identification and annotation

Single-nucleotide polymorphisms have been described in the Atlantic salmon IGF-1 gene promoter, which was discovered to be strongly linked with performance growth attributes (Tsai et al., 2015). A whole-transcriptome RNA-seq analysis from higher growth selected rainbow trout population versus unselected genetic cohorts revealed 22 cSNP markers associated with the growth rate (Salem et al., 2012). Around 5 million genomic SNPs from riverine population of rohu have been reported previously (Sahoo et al., 2022) along with 3,193 cSNPs for resistance against *Aeromonas hydrophila* in rohu along with the linkage map (Robinson et al., 2014). In this work, we have focused on identification of cSNPs in HB individuals to pinpoint the cSNPs associated with better performing individuals in terms of body growth. We could find around 39,158 high-quality cSNPs which harbored more transitions (A↔G or C↔T) than transversions (A↔C or T and G↔ C or T) and hinted at lesser protein changes attributed to cSNPs. Upon placement of SNPs in chromosomes, it was observed that maximum numbers (2,303) were present on chromosome 14, followed by chromosome 3 with 2,118 nos. and chromosome 19 with 1,671 cSNPs (Figure 7). A larger percent of cSNPs (9.5%) were present in the downstream region (3′ UTR) of genes as compared to the upstream located cSNPs (0.24%), implying that those located downstream may play a role in microRNA and post-transcriptional regulation of gene expression (Ali et al., 2019;Ali et al., 2019 2020). Fewer cSNPs found to be residing in the upstream gene region might be functional SNPs modulating binding of the transcription factor and changing the gene expression abilities (Li et al., 2018). Approximately 4,523 cSNPs were categorized as missense cSNPs, which are known to cause missense mutation. It is a type of non-synonymous substitution in which one amino acid is replaced with another, creating altered protein with functional and structural modifications, which might result in diseased conditions (Emadi et al., 2020). Further investigations on these cSNPs with differentially expressed transcripts are warranted to detect their contribution to better growth rate. Alternatively, 5,638 cSNPs were synonymous and considered silent effectors. A meager number (124) of cSNPs were classified as nonsense, which could possibly insert a stop codon into CDS and disrupt or truncate the formation of proteins (Chu & Wei, 2019). Of the significantly important ones, we could find two cSNPs in titin isoform X11, one of which was the splice donor variant, possibly playing role in splice site mutations, and other being missense. This type of mutation results in altered amino acid production due to change in codon sequence. We also noticed that significantly upregulated myogenic Factor 6 and insulin-like growth factor I isoform X1 had missense and upstream gene modifier SNP harboring within them, respectively. These two genes are located closely on the same chromosome No. 4 and are upregulated in the HB group. Another hotspot was detected on chromosome no. 14, which is also the highest SNP harboring chromosome in our study. Table 6 shows that this chromosome holds the eukaryotic translation initiation factor 4E-binding protein 3-like, transforming growth factor beta-2 proprotein, and meprin A subunit beta-like isoform X1 with 2, 1, and 1 cSNPs present within them, respectively. While the earlier ones are involved in cell differentiation and growth, the later gene, meprin A subunit beta-like, is situated away from both and is involved in cell proliferation (Schütte et al., 2010), cytokine processing, and inflammatory responses (Herzog, Haun, and Kaushal, 2019). The coding SNP present in meprin is annotated as a high-impact protein coding variant which might be responsible for its 3-fold upregulation. Furthermore, chromosome no. 19 harbored two predicted high-impact SNPs in thyroid hormone receptor beta isoform X1 and casein kinase II subunit beta. These genes are significantly upregulated in our studies and indicate their association with body weight in the HB group. Furthermore, the presence of immunity- and growth-related genes/SNPs in the vicinity on the same chromosome hints at the presence of possible QTLs, which needs to be validated in larger population for downstream applications such as marker panel development and association studies.

## 5 Conclusion

Overall results of this work established the substantial involvement of growth-related pathways such as GH/IGF growth axis, mTOR and proteolytic systems, and thyroid hormone regulation cascade toward body growth to which differential body weight between HB and LB groups is attributed. The transcriptome generated from genetically improved farmed rohu carp, “Jayanti”, in this study shall be a valuable genomic resource in expanding our knowledge on genetic architecture and expression dynamics of genes linked to harvest body weight and studying how genetic selection for economically important traits changes the transcription landscape. Furthermore, large-scale validation of cSNPs generated in this study may result in marker loci or candidate genes with possible QTL effects for use in marker-assisted breeding, SNP array construction for genome-wide association studies, and genomic selection for enhanced growth rate in improved rohu.

## Data Availability

The datasets presented in this study can be found in online repositories. The names of the repository/repositories and accession number(s) can be found in the article/Supplementary Material.

## Abbreviations

HB, high breeding value; LB, low breeding value; DEG, differentially expressed genes; EBV, estimated breeding value.
