# Supplementary material for: Identification of differentially expressed genes and SNPs linked to harvest body weight of genetically improved rohu carp, Labeo rohita
Source: Front Genet. 2023 Jun 8;14:1153911. doi: 10.3389/fgene.2023.1153911 (PMC10285081; doi:10.3389/fgene.2023.1153911)
Supplement: Supplementary file 1 [file DataSheet1.ZIP › Supplementry files_3revision/Supplementary Tables.docx]

**Table S1. Details of differentially expressed genes, primer sets and different parameters of RT-qPCR used for gene validation**

| **Gene name** | **Symbol** | **Function** | **Primer Sequence (5′ → 3′)** | **Size (bp)** | **Tm**  **(^0^C)** |
| --- | --- | --- | --- | --- | --- |
| Beta actin | β-actin | Cytoskeletal structural protein | F-CACTGTGCCCATCTACGAG | 184 | 58 |
|  |  |  | R-CCATCTCCTGCTCGAAGTC |  |  |
| Titin isoform X11 | Ttn | Elastic stabilization of myosin and actin filaments | F-ACGCTTTCGCTCTCTTCCAC | 182 | 58 |
|  |  |  | R-AAGGAGGGTCGTCCACAGAA |  |  |
| Acetyl CoA carboxylase Alpha | acaca | Regulation of fatty acid metabolism | F- ACTTGCCCTGCTTGTTGGAT | 135 | 57 |
|  |  |  | R- GACCCAGCGAATTTGGACTC |  |  |
| Plectin like isoform X4 | plctn | Myofibre integrity | F-ACTGGCGAAAGATGCGGAAAAG | 124 | 56 |
|  |  |  | R-AATTTGCTTGGCAGCTTCAGCC |  |  |
| Transforming Growth Factor beta 2 like | tgfb | Control of growth and proliferation of cells | F-TTTGCAGGCATTGACGACGAC | 115 | 59 |
|  |  |  | R-CAGTGGGCAGCAGGGTTAGT |  |  |
| Proheparin binding EGF like growth factor | LOC109081615 | Control of growth and proliferation of cells | F-CGCCCCACACCACTAAAACG | 185 | 58 |
|  |  |  | R-TCGTGAGTCAGGCCCTTCAC |  |  |
| Thyroid hormone receptor beta | thrb | Cell growth and differentiation | F-ACCGTAAGCACAAGGTGGCT | 173 | 59 |
|  |  |  | R-ACTGCTTGGCCGTCAGTCTT |  |  |
| Myogenic factor6 | myog | Muscle differentiation | GAGGCTGCCCAAAGTGGAGAT | 169 | 58 |
|  |  |  | GTCAGAGCGCATGGATCGGT |  |  |
| Insulin like growth factor 1 isoform 1 | LOC109085261 | Control of growth and proliferation of cells( mediator of the anabolic and mitogenic activity of GH) | TGCTGTTGTGTTGAGGAGGGT | 140 | 57 |
|  |  |  | CAGCGGCGATACCAAAAGCA |  |  |
| Fibroblast growth factor 4B like | fg4b | Cellular proliferation and differentiation | GGCATCCCCGTTTGGAACCT | 123 | 59 |
|  |  |  | ATAAGCTGCGGCCACTGACA |  |  |
| growth factor receptor-bound protein 10-like isoform X1 | LOC109076381 | Cellular proliferation and differentiation | CGCAGTGTGCTTTTCTCCAG | 126 | 56 |
|  |  |  | TAAACACCCCTGCACCACTG |  |  |
| Myostatin | mstn | Myoblast proliferation and differentiation | CCGAAGTCCTCGAAGTCCACA | 167 | 58 |
|  |  |  | AAACGCTGGCTCTTTGGTTTCT |  |  |
| ribosomal protein S6 kinase alpha-6 | Rps6 | Cellular growth and proliferation | TTAAGACGCCGAGAGACCACC | 166 | 58 |
|  |  |  | AGATGTGCTGCTGTGCCTTTTC |  |  |
| calpastatin-like isoform X9 | LOC109045535 | Endogenous regulation of calpain | GGGGTCAGTCAGGTTGGGAAT | 123 | 58 |
|  |  |  | GTAAAGCCCTCGGCACCTGA |  |  |
| unconventional myosin-1c isoform X1 | Myo1C | Microfilament motor activity | CGACCAATTGCCTTGCGGAT | 191 | 56 |
|  |  |  | GGTTTTCTGTTCATCCAAATGGAGC |  |  |
| Bromodomain-containing protein 3 | Brd3 | Regulation of transcription | GAGCGCCATTGATTTGTGAA | 124 | 54 |
|  |  |  | AGGGGGCAGATGGTAGGTTT |  |  |
| semaphorin-3aa isoform X1 | Sema3aa | Axon guidance/ Cellular morphology regulators | GTGCCTTCTGCGTGCTTTGA | 153 | 58 |
|  |  |  | AGAGAGCGCAAAACACACCC |  |  |

Table S2. RNA tapestation profile of libraries

| **Well** | **RIN** | **28S/18S (Area)** | **Conc. [ng/µl]** | **Sample Description** |
| --- | --- | --- | --- | --- |
| A1 | - | - | 125 | Ladder |
| B1 | 7.7 | 0.9 | 26.7 | RLBV.84 |
| C1 | 6.8 | 1.2 | 54.8 | RLBV82 |
| D1 | 8.2 | 0.7 | 223 | RLBV87 |
| E1 | 8.0 | 1.3 | 89.8 | RLBV89 |
| F1 | 8.0 | 1.3 | 39.5 | RLBV88 |
| G1 | 7.6 | 0.4 | 93.6 | RHBV104 |
| H1 | 7.7 | 1.0 | 49.2 | RHBV133 |
| A2 | 7.2 | 0.9 | 53.9 | RHBV108 |
| B2 | 7.5 | 1.2 | 25.4 | RHBV122 |
| C2 | 8.0 | 1.2 | 58.4 | RHBV121 |
| D2 | 7.7 | 1.0 | 31.0 | RHBV135 |

| **Transcript ID** | **Gene Symbol** | **logFC** | **Up/Down**  **Regulation** | **Adjusted P Value** | **Transcript Name** | **Function** |
| --- | --- | --- | --- | --- | --- | --- |
| TRINITY_DN1496_c0_g1_i4 | mical3a | 10.47 | Up | 2.66E-09 | protein-methionine sulfoxide oxidase mical3a isoform X1 | axonal growth cone repulsion, membrane trafficking, apoptosis |
| TRINITY_DN14795_c0_g2_i1 | LOC122134657 | 9.9 | Up | 2.15E-09 | AMP deaminase | Regulator of muscle energy metabolism |
| TRINITY_DN43_c5_g2_i31 | Phka1 | 9.38 | Up | 1.57E-08 | Phosphorylase b kinase regulatory subunit alpha | Involved in glycogen metabolism |
| TRINITY_DN6983_c0_g1_i11 | ACACA | 9.09 | Up | 8.22E-08 | acetyl-CoA carboxylase alpha | Regulation of fatty acid metabolism |
| TRINITY_DN18184_c0_g1_i10 | mical2b | 7.23 | Up | 1.01E-07 | protein-methionine sulfoxide oxidase mical2b-like | Membrane trafficking |
| TRINITY_DN29547_c0_g1_i6 | nuggc | 3.09 | Up | 3.40E-07 | Nuclear GTPase SLIP-GC | Downregulation of apoptosis |
| TRINITY_DN29428_c0_g1_i2 | LOC124030721 | 3.1 | Up | 3.91E-07 | serine/threonine-protein kinase SMG1-like | Role as mRNA-surveillance protein, key component of nonsense-mediated decay (NMD) |
| TRINITY_DN164_c0_g1_i6 | Csnk2b | 10.15 | Up | 4.11E-07 | casein kinase II subunit beta | regulates metabolic pathways, signal transduction, transcription, translation, and replication |
| TRINITY_DN5709_c0_g1_i4 | LOC113049299 | 9.76 | Up | 7.96E-07 | endoplasmic reticulum mannosyl-oligosaccharide 1,2-alpha-mannosidase-like | Misfolded glycoproteins targeted for degradation by glycoprotein quality control |
| TRINITY_DN43_c5_g2_i38 | LOC109094309 | 9.67 | Up | 1.18E-06 | phosphorylase b kinase regulatory subunit alpha, skeletal muscle isoform-like | Regulator of cell growth |
| TRINITY_DN12932_c0_g1_i1 | LOC109050849 | 9.23 | Up | 1.14E-06 | phosphatase 1 regulatory subunit 3A-like isoform X4 | Regulation of glycogen metabolism |
| \| TRINITY_DN7737_c0_g1_i16 \| \| --- \| \|  \| | LOC109084930 | 8.2 | Up | 1.12E-06 | LINE-1 reverse transcriptase -like protein | Cell proliferation and differentiation |
| TRINITY_DN19381_c1_g1_i6 | *ppef2* | 7.2 | Up | 1.28E-06 | serine/threonine-protein phosphatase with EF-hands 2-like | Phosphoprotein phosphatase activity |
| TRINITY_DN7508_c0_g2_i10 | LOC113118243 | 6.97 | Up | 2.93E-06 | malonyl-CoA decarboxylase, mitochondrial-like | Regulation of fatty acid production and utlilization |
| TRINITY_DN7002_c0_g1_i11 | Brd2 | 10.9 | Up | 4.76E-08 | Bromodomain-containing protein 2 | Signal transduction pathway involved in growth |
| TRINITY_DN3688_c2_g1_i2 | LOC109105691 | 8.5 | Up | 4.58E-06 | cathepsin D-like | Signal transduction pathway involved in growth |
| TRINITY_DN433_c0_g1_i15 | eif3c | 7.35 | Up | 5.81E-06 | eukaryotic translation initiation factor 3 subunit C isoform X1 | translation initiation factor activity |
| TRINITY_DN59658_c0_g2_i1 | tgfb5 | 7.33 | Up | 9.04E-06 | transforming growth factor beta-2-like | Control of growth and proliferation of cells |
| TRINITY_DN1686_c0_g1_i3 | [LOC113113723](https://www.ncbi.nlm.nih.gov/gene/113113723) | 7.07 | Up | 9.53E-06 | proheparin-binding EGF-like growth factor | Control of growth and proliferation of cells |
| TRINITY_DN64976_c1_g1_i2 | Thrb | 7.01 | Up | 1.07E-05 | thyroid hormone receptor beta | Cell growth and differentiation |
| TRINITY_DN94989_c0_g1_i6 | eogt | 6.90 | Up | 1.27E-05 | EGF domain-specific O-linked N-acetylglucosamine transferase-like | protein N-acetylglucosaminyltransferase activity |
| TRINITY_DN326_c27_g1_i2 | LOC113110788 | 6.29 | Up | 1.34E-05 | myomesin-2-like | skeletal muscle development |
| TRINITY_DN51246_c0_g1_i3 | SPEG | 7.37 | Up | 1.57E-05 | putative striated muscle preferentially expressed protein kinase-like protein | Regulator of muscle development |
| TRINITY_DN1096_c0_g1_i3 | [LOC109112830](https://www.ncbi.nlm.nih.gov/gene/109112830) | 7.36 | Up | 1.53E-05 | tensin-1 isoform X3 | Muscle regeneration |
| TRINITY_DN42096_c0_g1_i4 | LOC113105794 | 7.3 | Up | 1.78E-05 | zinc finger BED domain-containing protein 1-like | Protein modification |
| TRINITY_DN47475_c1_g1_i3 |  | 7.3 | Up | 2.30E-05 | activin receptor type-2A-like isoform X2 | Control of myostatin/Activin A complex |
| TRINITY_DN23824_c0_g2_i1 | [LOC109059739](https://www.ncbi.nlm.nih.gov/gene/109059739) | 3.0 | Up | 2.56E-05 | calpain small subunit 1-like | Involved in apoptotic control, migration, adhesion and autophagy |
| TRINITY_DN3483_c2_g1_i21 | LOC109111554 | 3.5 | Up | 3.41E-05 | calpain-2 catalytic subunit-like | Cytoskeletal remodelling |
| TRINITY_DN2181_c0_g1_i3 | LOC114868025 | 2.6 | Up | 3.37E-05 | calpain-5-like | Muscle texture |
| TRINITY_DN11027_c0_g1_i2 | RIO2 | 2.42 | Up | 3.50E-05 | serine/threonine-protein kinase RIO2 | Involved in PI3K/AKT pathways |
| TRINITY_DN30192_c0_g1_i30 | Wnk4 | 2.26 | Up | 3.56E-05 | serine/threonine-protein kinase WNK4 | Involved in PI3K/AKT pathways |
| TRINITY_DN324_c5_g1_i13 | LOC10dy9071685 | 2.1 | Up | 4.47E-05 | Calpain 3-like | Sarcomere remodelling |
| TRINITY_DN19323_c0_g1_i3 | LOC113040912 | 2.34 | Up | 4.53E-05 | cyclin-dependent kinase 12-like isoform X1 | Cell cycle progression |
| TRINITY_DN4032_c3_g1_i7 | LOC109102249 | 2.75 | Up | 4.90E-05 | transmembrane protein 255A-like isoform X2 | Molecular transport |
| TRINITY_DN1911_c4_g1_i8 | LOC113118389 | 3.4 | Up | 6.08E-05 | cortactin-binding protein 2-like | Stabilization of actin filaments |
| TRINITY_DN10439_c1_g1_i14 | GRB10 | 2.93 | Up | 7.36E-05 | growth factor receptor-bound protein 10-like isoform X1 | Cell growth and differentiation |
| TRINITY_DN3673_c0_g1_i5 | LOC121284791 | -3.36 | Dn | 8.92E-05 | myc box-dependent-interacting protein 1-like isoform X1 | Regulation of cell cycle |
| TRINITY_DN368_c0_g1_i4 | LOC109069805 | -5.90 | Dn | 9.56E-05 | tubulin alpha-8 chain-like | Mitotic cell cycle |
| TRINITY_DN29816_c0_g1_i10 | Mef2cb | -6.61 | Dn | 1.08E-04 | myocyte enhancer factor 2cb isoform X2 | skeletal muscle development |
| TRINITY_DN12982_c0_g1_i19 | [LOC109056862](https://www.ncbi.nlm.nih.gov/gene/109056862) | -7.3 | Dn | 1.44E-04 | ribosomal protein S6 kinase alpha-6 | Regulated by growth factors |
| TRINITY_DN10214_c1_g2_i18 | phldb1b | 7.87 | Up | 1.57E-04 | pleckstrin homology-like domain family B member 1 | Interaction with growth factor regulated protein kinase B |
| TRINITY_DN177_c0_g1_i20 | LOC113063923 | -7.8 | Dn | 1.64E-04 | calpastatin-like isoform X9 | Calpain inhibitor |
| TRINITY_DN296727_c0_g1_i1 | LOC109071423 | -7.9 | Dn | 1.79E-04 | striated muscle preferentially expressed protein kinase-like | Growth and differentiation of muscle cells |
| TRINITY_DN7002_c0_g1_i4 | Brd2 | -9.6 | Dn | 1.96E-04 | Bromodomain-containing protein 2 | Signal transduction pathway involved in growth |
| TRINITY_DN21494_c11_g1_i1 | NA | -9.9 | Dn | 2.07E-04 | No Hit |  |
| TRINITY_DN5068_c1_g1_i3 | Sema3aa | -10.0 | Dn | 2.03E-04 | semaphorin-3aa isoform X1 | Regulation of cell migration |
| TRINITY_DN31535_c0_g1_i2 | recg | -10.263622 | Dn | 2.02E-04 | ATP-dependent DNA helicase RecG | DNA replication and repair |
| TRINITY_DN22935_c4_g1_i14 |  | -10.813283 | Dn | 2.12E-04 | No Hit |  |
| TRINITY_DN159785_c0_g1_i2 | Ubxn4 | -11.155884 | Dn | 2.22E-04 | UBX domain-containing protein 4 | Involved in endoplasmic reticulum-associated protein degradation (ERAD). |
| TRINITY_DN901_c110_g1_i1 | [LOC113051792](https://www.ncbi.nlm.nih.gov/gene/113051792) | -2.69 | Dn | 2.43E-04 | anoctamin-1-like isoform X2 | glucose-stimulated insulin secretion |
| TRINITY_DN19891_c0_g2_i1 | LOC113091087 | -3.4 | Dn | 2.57E-04 | prominin-1-A-like isoform X5 | Determination of cellular morphology |
| TRINITY_DN82_c4_g2_i2 | [LOC113046573](https://www.ncbi.nlm.nih.gov/gene/113046573) | 7.1 | Up | 2.56E-04 | myosin-binding protein C, fast-type-like isoform X1 | Skeletal muscle contraction |
| TRINITY_DN385_c4_g1_i7 | LOC109106330 | 6.1 | Up | 2.61E-04 | unconventional myosin-Va-like | Intracellular trafficking and transcription |
| TRINITY_DN39814_c0_g2_i4 | LOC109105750 | 7.1 | Up | 2.71E-04 | myosin-10-like isoform X6 | cytokinesis, cell motility, and cell polarity |
| TRINITY_DN1894_c13_g1_i11 | [LOC113069116](https://www.ncbi.nlm.nih.gov/gene/113069116) | 6.7 | Up | 3.05E-04 | myosin-7-like | Skeletal muscle contraction |
| TRINITY_DN846_c0_g1_i7 | LOC109097713 | 8 | Up | 3.27E-04 | unconventional myosin-XVIIIb-like | Muscle integrity |
| TRINITY_DN78515_c1_g1_i1 | [LOC109062378](https://www.ncbi.nlm.nih.gov/gene/109062378) | 2.6 | Up | 3.22E-04 | myosin-9-like | Cytokinesis and signal transduction |
| TRINITY_DN369_c1_g1_i8 | obscnb | 8.72 | Up | 3.34E-04 | obscurin isoform X17 | Organization of myofibrils |
| TRINITY_DN499_c0_g1_i17 | LOC109054846 | 2.35 | Up | 4.41E-04 | spectrin alpha chain, non-erythrocytic 1 isoform X1 | Cell cycle regulation |
| TRINITY_DN121_c3_g1_i1 | LOC10907573 | 9.38 | Up | 4.35E-04 | dystrobrevin beta-like isoform X1 | Scaffolding / intracellular signal transduction |
| TRINITY_DN24420_c0_g1_i12 | LOC113042581 | 7.26 | Up | 4.73E-04 | dystrobrevin alpha-like isoform X1 | Scaffolding / intracellular signal transductionsp |
| TRINITY_DN273_c0_g1_i262 | Ttn | 9.11 | Up | 4.88E-04 | titin isoform X11 | Elastic stabilization of myosin and actin filaments |
| TRINITY_DN711_c0_g1_i6 | [LOC109078612](https://www.ncbi.nlm.nih.gov/gene/109078612) | 2.1 | Up | 5.02E-04 | tropomyosin alpha-4 chain isoform X3 | Filament stability |
| TRINITY_DN6516_c0_g1_i1 | [LOC109111053](https://www.ncbi.nlm.nih.gov/gene/109111053) | 4.1 | Up | 2.84E-03 | tropomyosin alpha-3 chain isoform X7 | Filament stability |
| TRINITY_DN10514_c0_g1_i2 | LOC113119167 | 2.7 | Up | 5.07E-04 | unconventional myosin-IXb-like isoform X1 | Muscle integrity |
| TRINITY_DN14078_c0_g1_i12 | LOC109105750 | 5.7 | Up | 5.32E-04 | myosin-10-like isoform X1 | cytokinesis, cell motility, and cell polarity |
| TRINITY_DN1023_c3_g1_i33 | LOC113120112 | 8.70 | Up | 5.25E-04 | plectin-like isoform X4 | Myofibre integrity |
| TRINITY_DN5215_c0_g1_i42 | LOC122134417 | 8.69 | Up | 5.46E-04 | nebulin-like isoform X1 | Thin filament length specification |
| TRINITY_DN39644_c1_g1_i8 | LOC118821686 | -4.3 | Dn | 5.49E-04 | myosin-1 isoform X3 | Cell adhesion, actin architecture |
| TRINITY_DN294_c25_g2_i2 | LOC113092685 | -2.2 | Dn | 6.34E-04 | actin, cytoplasmic 1 | Muscle contraction and cell movement |
| TRINITY_DN1612_c6_g1_i1 | [actc1a](https://www.ncbi.nlm.nih.gov/gene/408256) | 3.32 | Up | 6.39E-04 | actin, alpha cardiac muscle 1-like | Muscle contraction and cell movement |
| TRINITY_DN2498_c6_g1_i6 | LOC106567840 | -8.13 | Dn | 6.57E-04 | unconventional myosin-Ic isoform X1 | Calmodulin binding |
| TRINITY_DN104883_c2_g1_i5 | [LOC109077262](https://www.ncbi.nlm.nih.gov/gene/109077262) | 3.42 | Up | 7.50E-04 | proline-serine-threonine phosphatase-interacting protein 1-like | Immunity functions |
| TRINITY_DN27501_c0_g1_i3 | raraa | 8.5 | Up | 7.55E-04 | retinoic acid receptor alpha | Aids in promoting myogenesis by antagonizing TGFbeta signaling |
| TRINITY_DN11685_c0_g1_i4 | [LOC113079191](https://www.ncbi.nlm.nih.gov/gene/113079191) | 2.5 | Up | 7.83E-04 | myocyte-specific enhancer factor 2C-like isoform X1 | myocyte differentiation and hypertrophic gene expression |
| TRINITY_DN11685_c0_g1_i9 | Mef2ca | 5.0 | Up | 8.19E-04 | myocyte-specific enhancer factor 2C | myocyte differentiation and hypertrophic gene expression |
| TRINITY_DN3606_c2_g1_i4 | Myf6 | 7.01 | Up | 9.98E-04 | Myogenic factor 6 | Muscle differentiation |
| TRINITY_DN6862_c2_g1_i10 | NO id | 7.36 | Up | 1.03E-03 | SPT6H factor | Promotes the activation of the myogenic gene program by entailing erasure of the repressive H3K27me3 epigenetic mark through stabilization of the chromatin interaction of the H3K27 demethylase KDM6A |
| TRINITY_DN4625_c0_g1_i4 | SIGLEC6 | 2.27 | Up | 1.03E-03 | sialic acid-binding Ig-like lectin 6 isoform X1 | Cell-cell interactions |
| TRINITY_DN471_c0_g1_i18 | LOC119501467 | 7.00 | Up | 1.02E-03 | Interleukin enhancer-binding factor 3 homolog isoform X2 | Regulation of immunity and growth |
| TRINITY_DN63283_c0_g1_i8 | hsp70 | 4.4 | Up | 1.02E-03 | Hsp70 family protein | Regulator of immune response |
| TRINITY_DN38629_c0_g1_i3 | LOC109103381 | 2.36 | Up | 1.11E-03 | Apolipoprotein L6-like isoform X1 | Regulation of apoptosis |
| TRINITY_DN29881_c0_g1_i3 | LOC109058439 | 2.15 | Up | 1.16E-03 | 14 kDa apolipoprotein | Innate immune system |
| TRINITY_DN14545_c3_g1_i1 | LOC109068604 | 2.61 | Up | 1.18E-03 | Apolipo L3-like protein | Bactericidal properties |
| TRINITY_DN47818_c0_g1_i1 | LOC109097353 | -2.3 | Dn | 1.19E-03 | Septin-5-like isoform X2 | Inflammation control |
| TRINITY_DN10363_c0_g1_i1 | LOC109064888 | -7.1 | Dn | 1.19E-03 | Septin-2B isoform X1 | Regulation of homeostasis |
| TRINITY_DN4366_c1_g2_i1 | septin2 | 3.6 | Up | 1.19E-03 | Septin 2 | Regulation of homeostasis |
| TRINITY_DN4913_c0_g1_i2 | LOC109053681 | 3.9 | Up | 1.19E-03 | Heat shock protein HSP 90-alpha | Aids in protein degradation |
| TRINITY_DN2802_c1_g1_i1 | NO id | -2.1 | Dn | 2.89E-03 | HSP20-like chaperone | Oxidative stress |
| TRINITY_DN226_c0_g1_i11 | Hsp90aa1.1 | 6.8 | Up | 1.20E-03 | Heat shock protein HSP 90-alpha 1 | Aids in protein degradation |
| TRINITY_DN1928_c2_g1_i2 | LOC109060497 | -2.1 | Dn | 1.22E-03 | L-rhamnose-binding lectin CSL2-like | Carbohydrate recognition |
| TRINITY_DN3831_c1_g1_i4 | thbs4b | 2.52 | Up | 1.41E-03 | Thrombospondin-4-B-like isoform X1 | Controls vascular inflammation |
| TRINITY_DN10630_c4_g1_i2 | Ighv | 5.9 | Up | 1.41E-03 | Immunoglobulin zeta heavy chain |  |
| TRINITY_DN19820_c1_g1_i1 | NA | 2.1 | Up | 1.42E-03 | Immunoglobulin heavy chain variable region | Antigen binding properties |
| TRINITY_DN30192_c0_g1_i30 | LOC109068174 | 2.27 | Up | 1.46E-03 | Polymeric immunoglobulin receptor-like protein | transcytosis of igA, immunoglobulin M (pIg) and immune complexes |
| TRINITY_DN43356_c0_g1_i6 | LOC109083095 | 2.37 | Up | 1.59E-03 | Immunoglobulin-like and fibronectin type III domain-containing protein 1 | homophilic cell adhesion |
| TRINITY_DN64112_c0_g1_i4 | [LOC109068174](https://www.ncbi.nlm.nih.gov/gene/109068174) | 6.1 | Up | 1.67E-03 | Polymeric immunoglobulin receptor-like | Transcytosis of immune complexes |
| TRINITY_DN88680_c1_g1_i9 | LOC109109090 | -6.8 | Dn | 1.66E-03 | Immunoglobulin-like domain-containing receptor 2 isoform X1 | T cell immune checkpoint |
| TRINITY_DN192197_c2_g1_i1 | LOC109105203 | 2.48 | Up | 1.69E-03 | pentraxin-related protein PTX3-like isoform X2 | Regulation of inflammation |
| TRINITY_DN378_c0_g2_i2 | LOC109084427 | 6.93 | Up | 1.83E-03 | prohibitin-2-like | Inner membrane mitophagy receptor |
| TRINITY_DN92617_c0_g1_i3 | LOC109059765 | -2.6 | Dn | 1.91E-03 | liver-expressed antimicrobial peptide 2-like (leap2 like) | modulatory effector of innate immunity |
| TRINITY_DN2793_c1_g1_i1 | LOC109103244 | 2.69 | Up | 1.94E-03 | tapasin-related protein-like | maturation of MHC class I molecules |
| TRINITY_DN4695_c0_g1_i6 | [LOC109069624](https://www.ncbi.nlm.nih.gov/gene/109069624) | 2.69 | Up | 1.93E-03 | macrophage mannose receptor 1-like | pattern-recognition receptokinr |
| TRINITY_DN8039_c1_g1_i4 | LOC109099030 | 3.63 | Up | 1.92E-03 | macrophage receptor MARCO-like | Improved phagocytic mechanism |
| TRINITY_DN1109_c6_g1_i2 | LOC122146951 | 6.5 | Up | 1.95E-03 | Mucin 19 like | Modulation of mucus secretion |
| TRINITY_DN8056_c0_g1_i16 | Tcf25 | 6.44 | Up | 1.97E-03 | transcription factor 25 | Control of apoptosis |
| TRINITY_DN305610_c0_g1_i1 | HIVEP2 | 3.06 | Up | 2.11E-03 | transcription factor HIVEP2-like | Control of gene expression |
| TRINITY_DN280183_c0_g2_i1 | cusR | -2.1 | Dn | 2.12E-03 | copper response regulator transcription factor CusR | Involved in phosphorelay signal transduction system |
| TRINITY_DN193788_c0_g1_i4 | [LOC109103179](https://www.ncbi.nlm.nih.gov/gene/109103179) | 2.43 | Up | 2.39E-03 | myb-related transcription factor, partner of profilin-like | Antiviral protein |
| TRINITY_DN142477_c0_g1_i2 | *Rest* | 6.3 | Up | 2.39E-03 | RE1-silencing transcription factor | Neural cell development |
| TRINITY_DN109373_c0_g2_i3 |  | 7.26 | Up | 2.37E-03 | Paired box protein Pax-3b | Cell proliferation and regulation |
| TRINITY_DN105949_c0_g1_i2 | LOC109058210 | -5.95 | Dn | 2.35E-03 | transcription factor IIIB 90 kDa subunit-like | General activator of RNA polymerase |

|  |  |  |  |  |
| --- | --- | --- | --- | --- |
|  |  |  |  |  |
|  |  |  |  |  |
|  |  |  |  |  |
|  |  |  |  |  |
|  |  |  |  |  |
|  |  |  |  |  |
|  |  |  |  |  |
|  |  |  |  |  |
|  |  |  |  |  |
|  |  |  |  |  |
|  |  |  |  |  |
|  |  |  |  |  |
|  |  |  |  |  |
|  |  |  |  |  |
|  |  |  |  |  |
|  |  |  |  |  |
|  |  |  |  |  |
|  |  |  |  |  |
|  |  |  |  |  |
|  |  |  |  |  |
|  |  |  |  |  |

**Table 3**. List of top significantly up and down regulated transcripts and their functions.
