# Supplementary figures and images for: Identification of differentially expressed genes and SNPs linked to harvest body weight of genetically improved rohu carp, Labeo rohita
Source: Front Genet. 2023 Jun 8;14:1153911. doi: 10.3389/fgene.2023.1153911 (PMC10285081; doi:10.3389/fgene.2023.1153911)

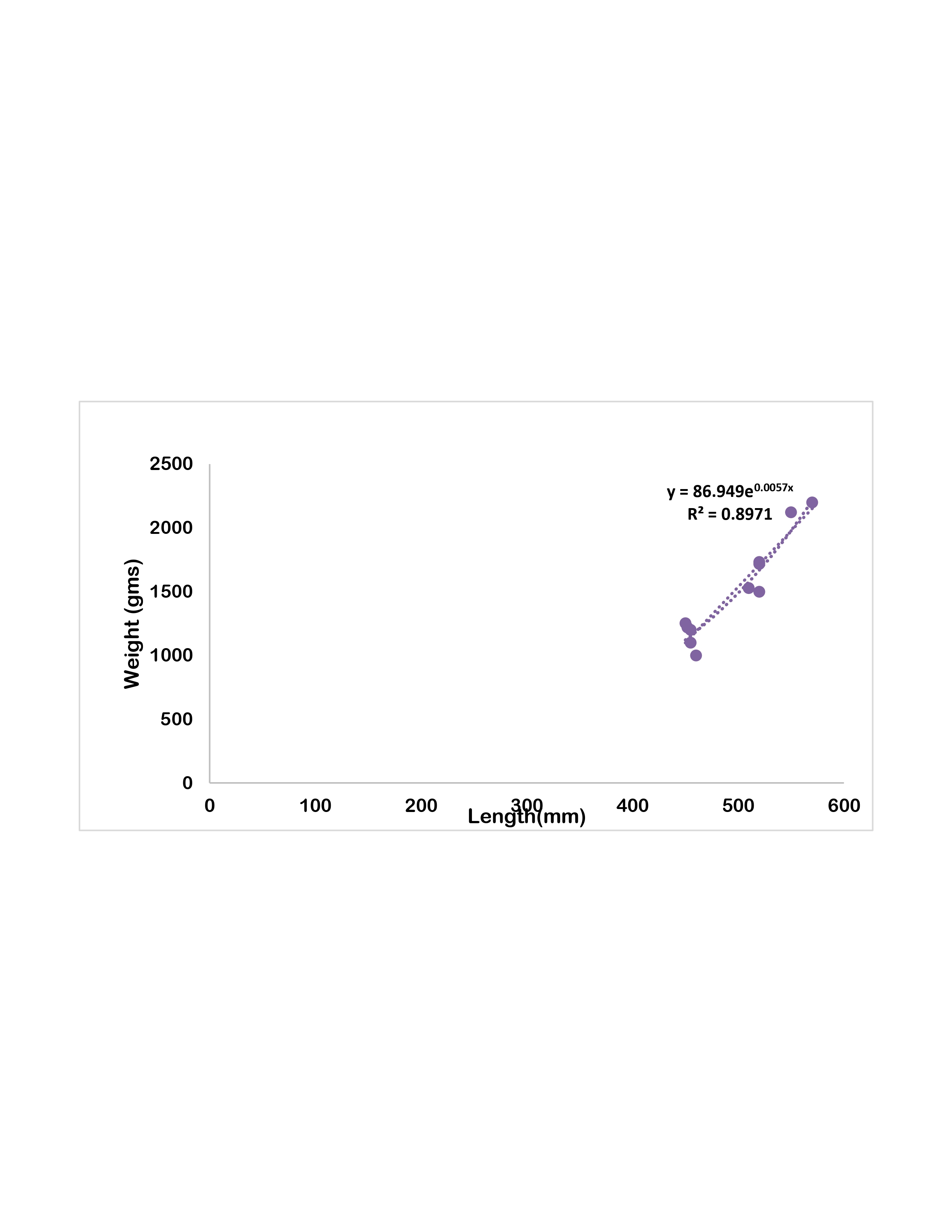

Supplement: Supplementary file 1 [file DataSheet1.ZIP › Supplementry files_3revision/Fig S1_Length Weight relationship of sampled individuals.jpg]

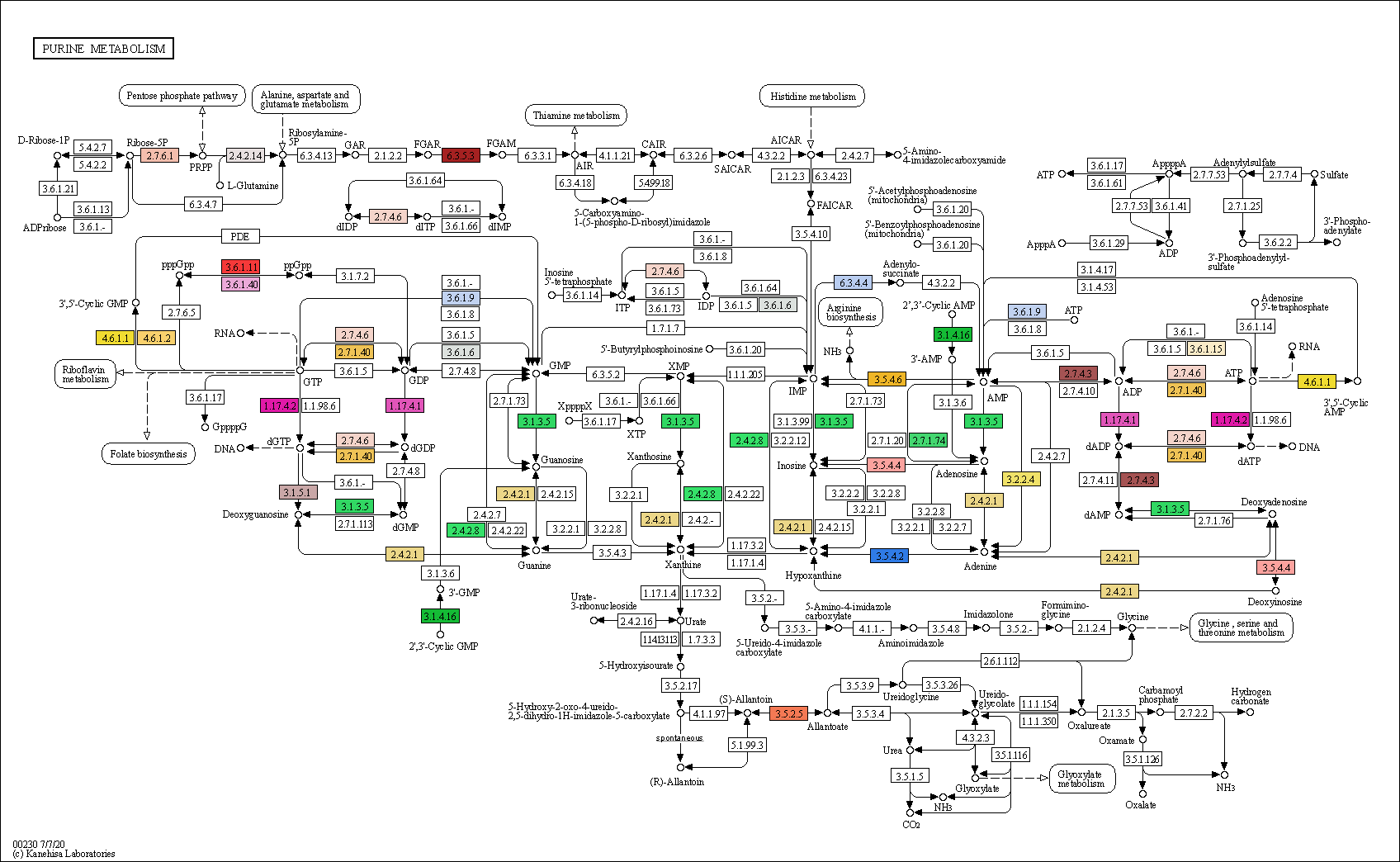

Supplement: Supplementary file 1 [file DataSheet1.ZIP › Supplementry files_3revision/Fig S10 Purine metabolism.jpeg]

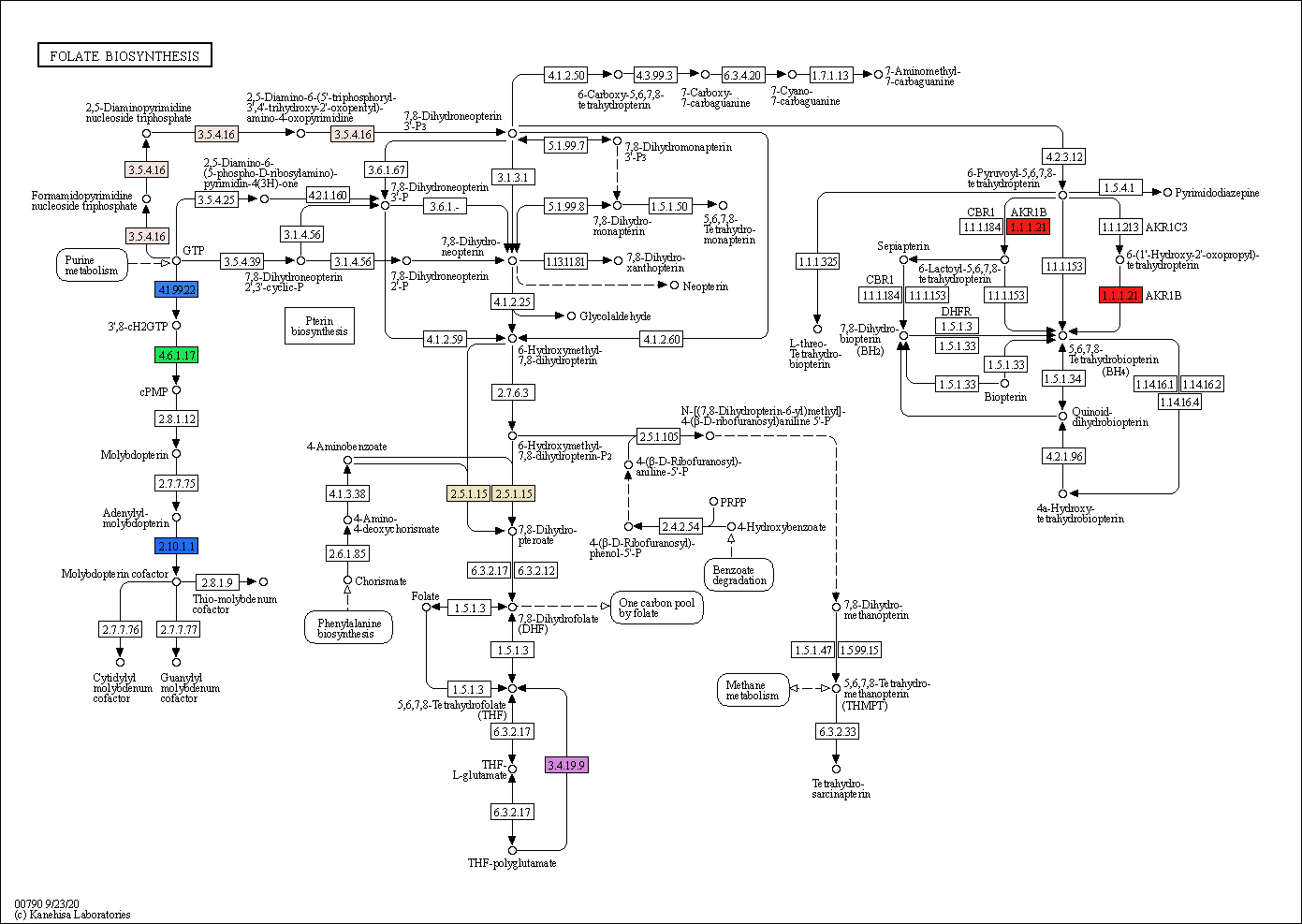

Supplement: Supplementary file 1 [file DataSheet1.ZIP › Supplementry files_3revision/Fig S11 Folate biosynthesis.jpeg]

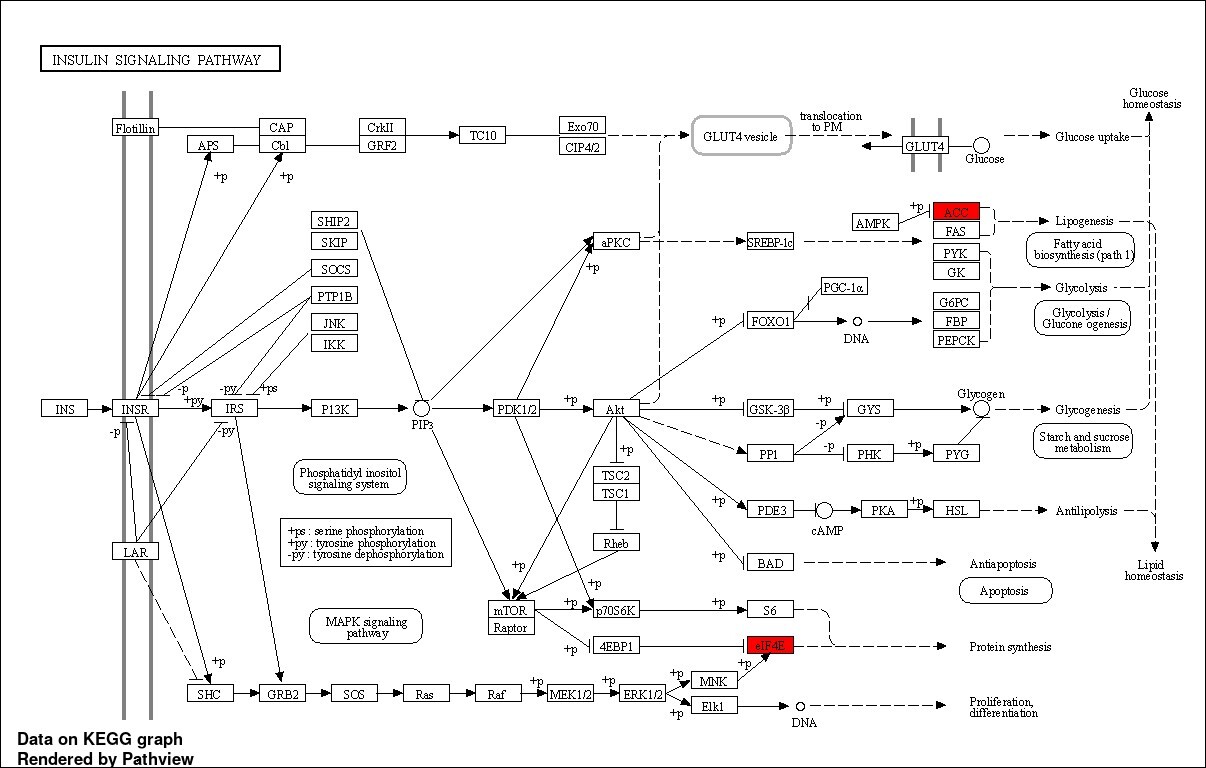

Supplement: Supplementary file 1 [file DataSheet1.ZIP › Supplementry files_3revision/Fig S12 insulin pathway.jpg]

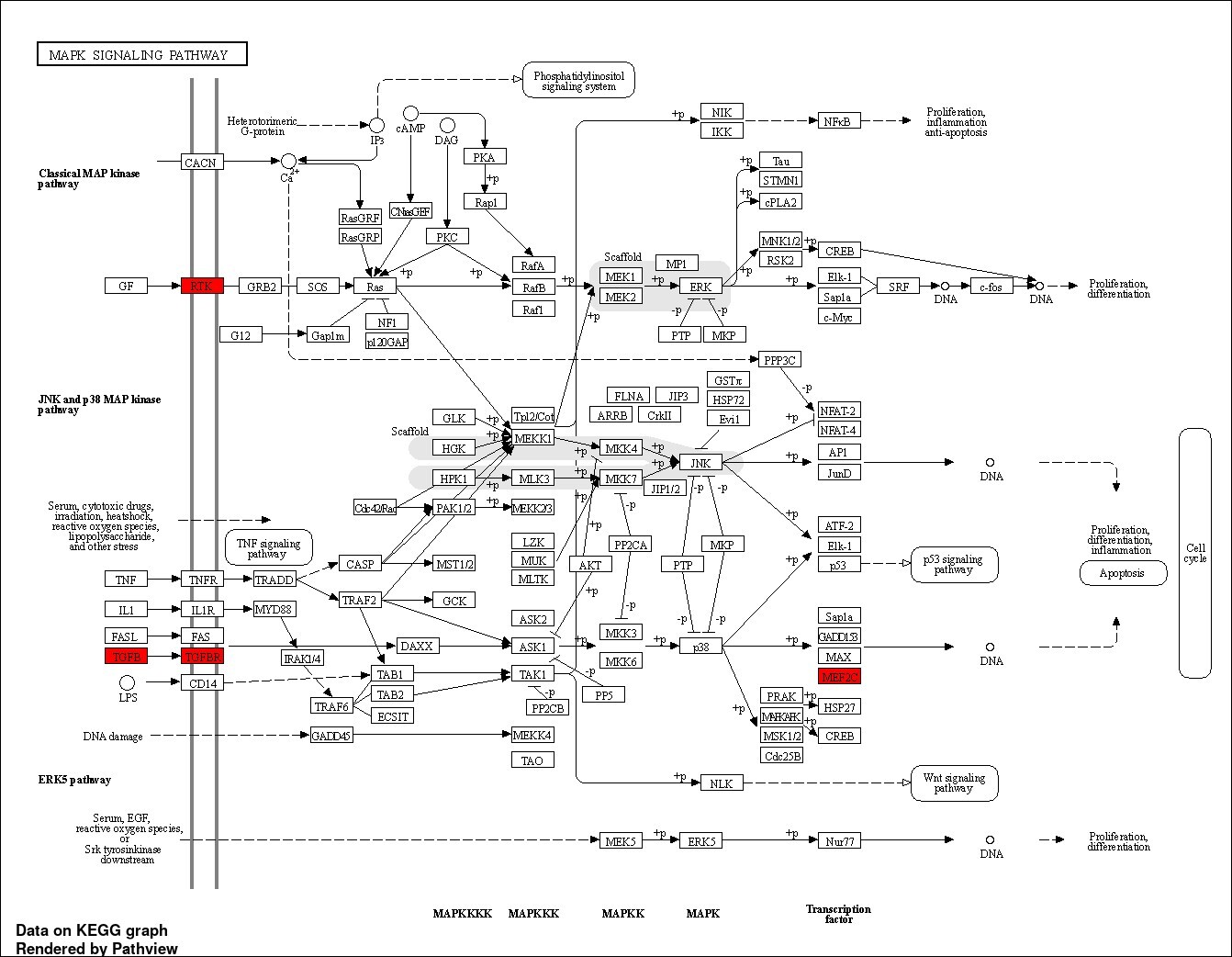

Supplement: Supplementary file 1 [file DataSheet1.ZIP › Supplementry files_3revision/Fig S13 MAPK pathway.jpg]

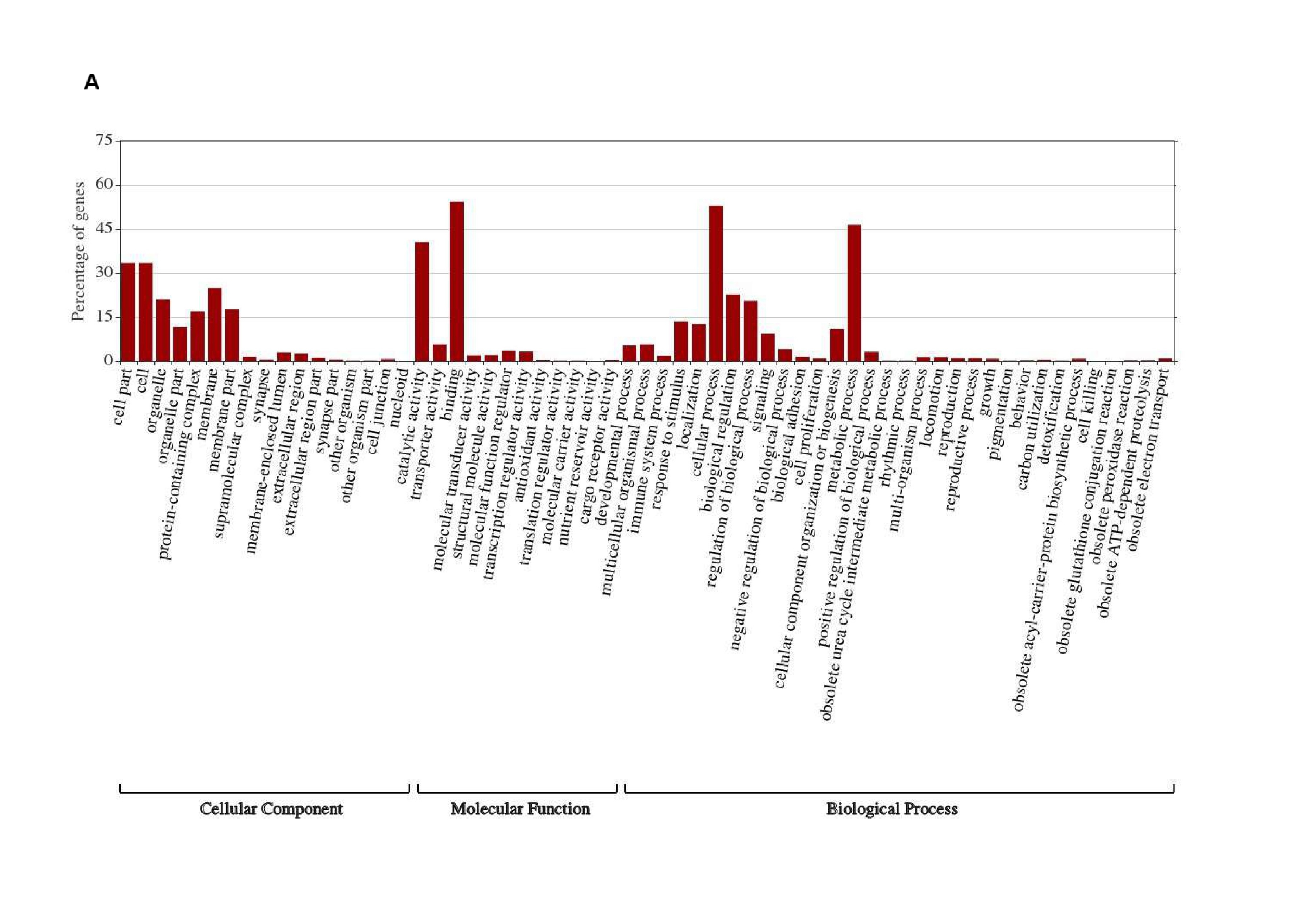

Supplement: Supplementary file 1 [file DataSheet1.ZIP › Supplementry files_3revision/Fig S2_Functional annotation of Differentially Expressed Transcripts (DETs).jpg]

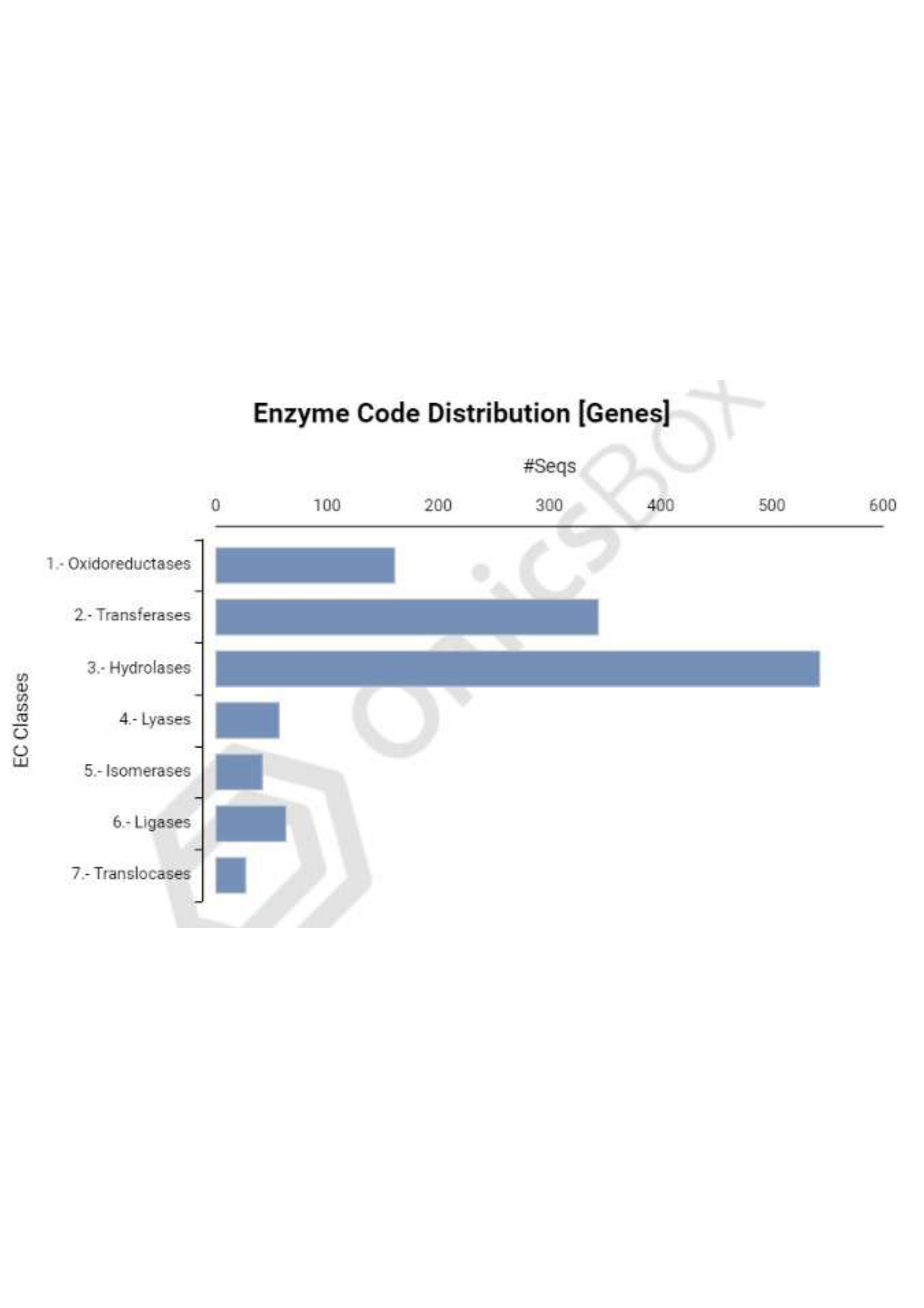

Supplement: Supplementary file 1 [file DataSheet1.ZIP › Supplementry files_3revision/Fig S3_ Enzyme code distribution.jpg]

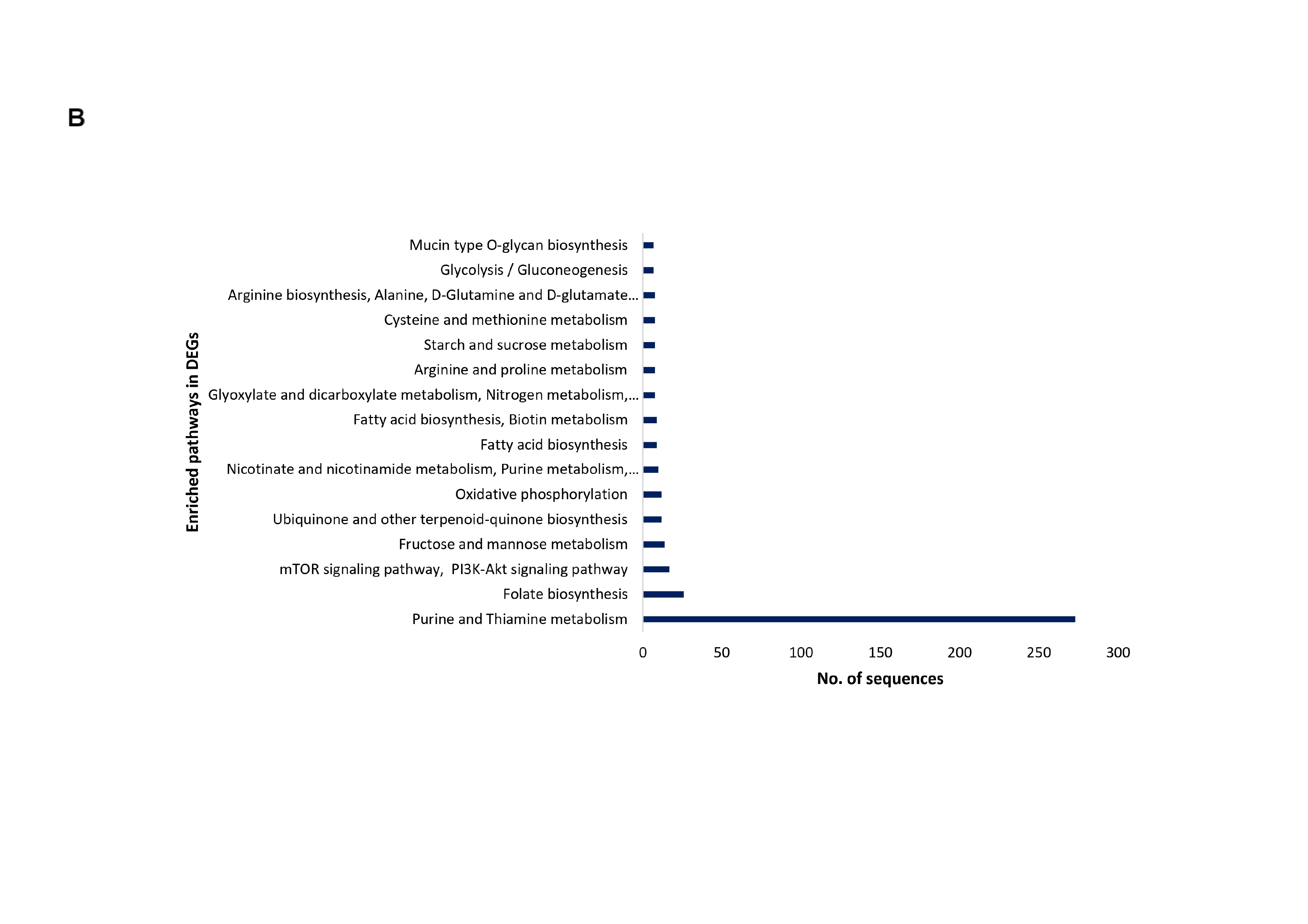

Supplement: Supplementary file 1 [file DataSheet1.ZIP › Supplementry files_3revision/Fig S4_Enriched KEGG pathways.jpg]

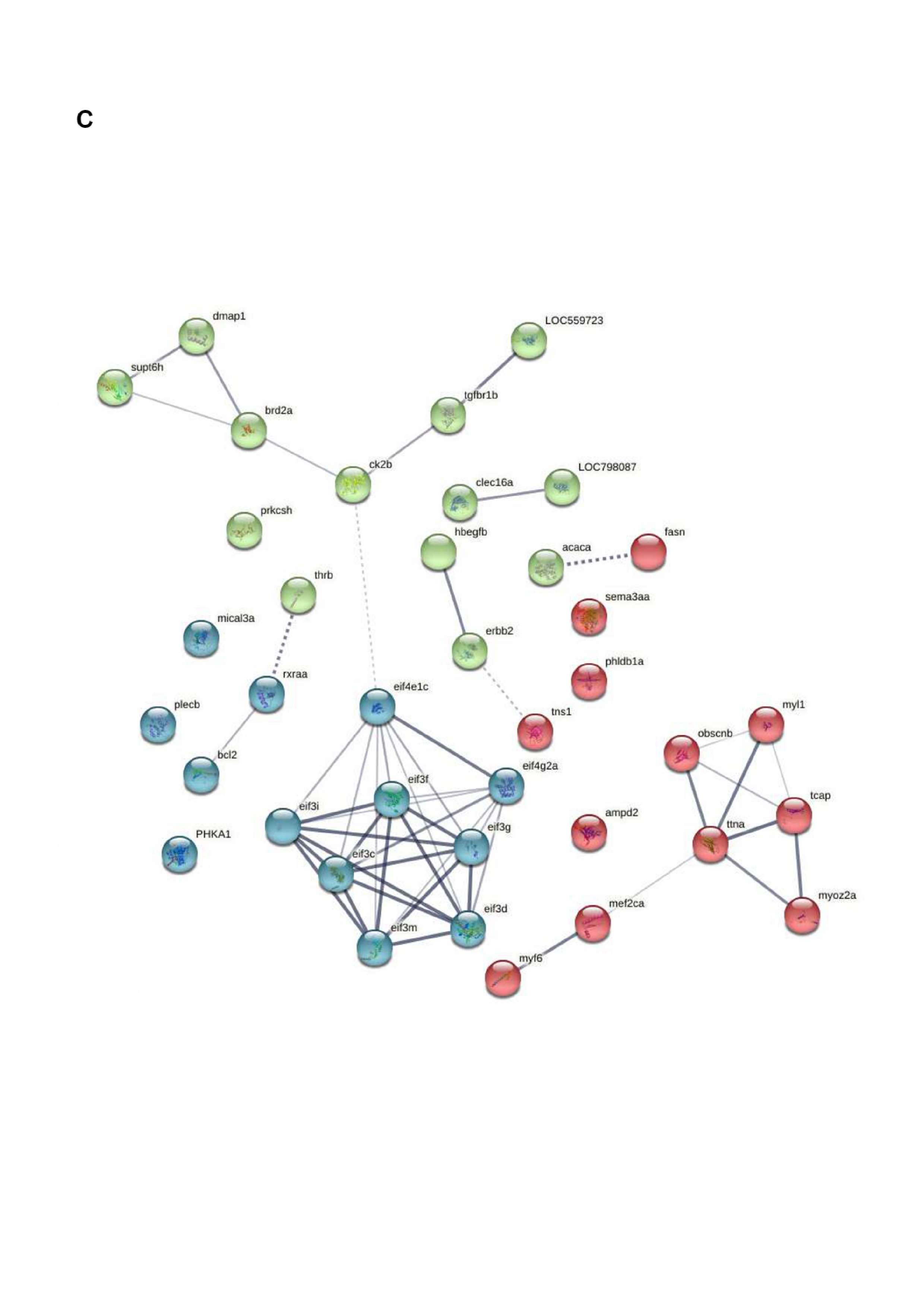

Supplement: Supplementary file 1 [file DataSheet1.ZIP › Supplementry files_3revision/Fig S5_Protein Protein Interaction (PPI) network for Differentially Expressed Gene clusters.jpg]

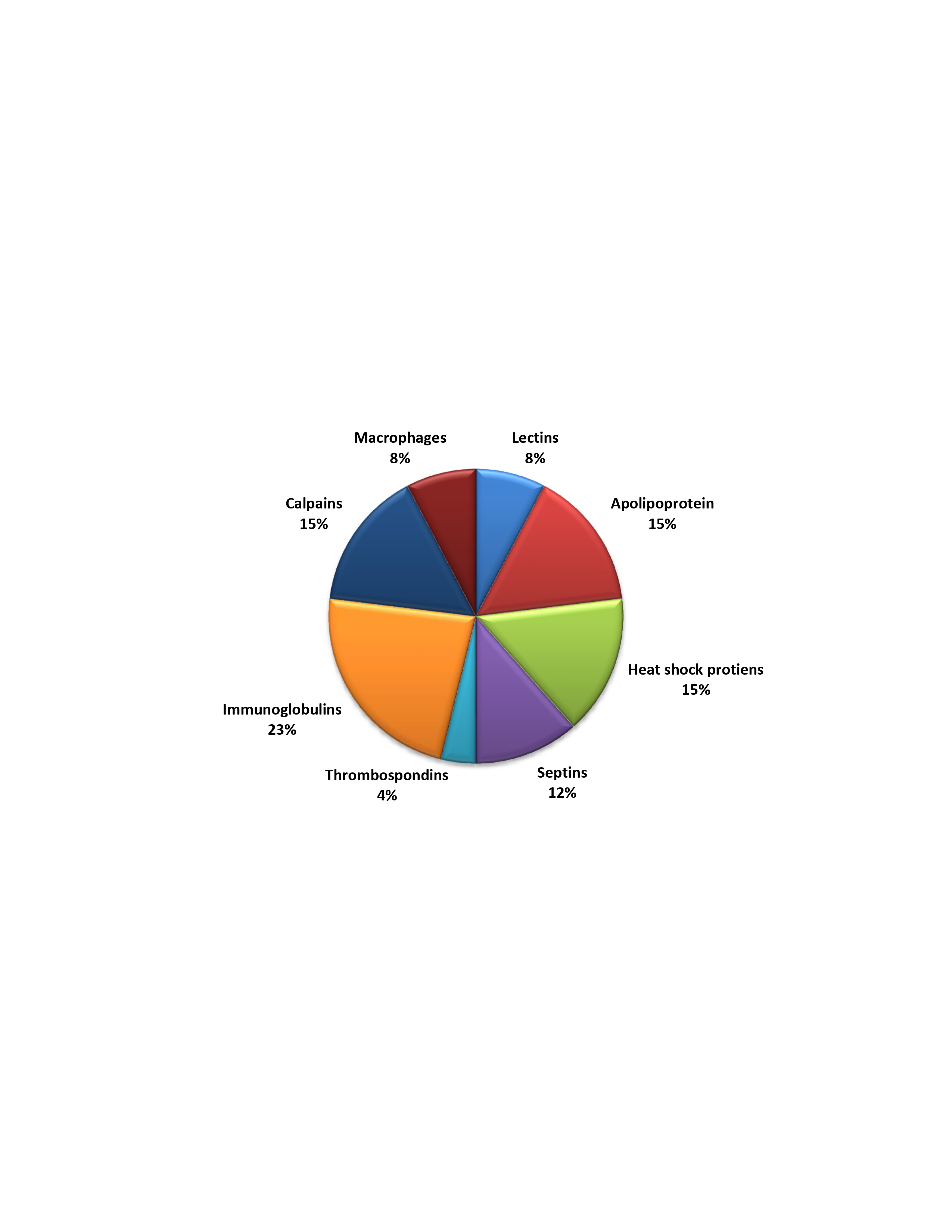

Supplement: Supplementary file 1 [file DataSheet1.ZIP › Supplementry files_3revision/Fig S6_Distribution of immunity genes identified from DETs.jpg]

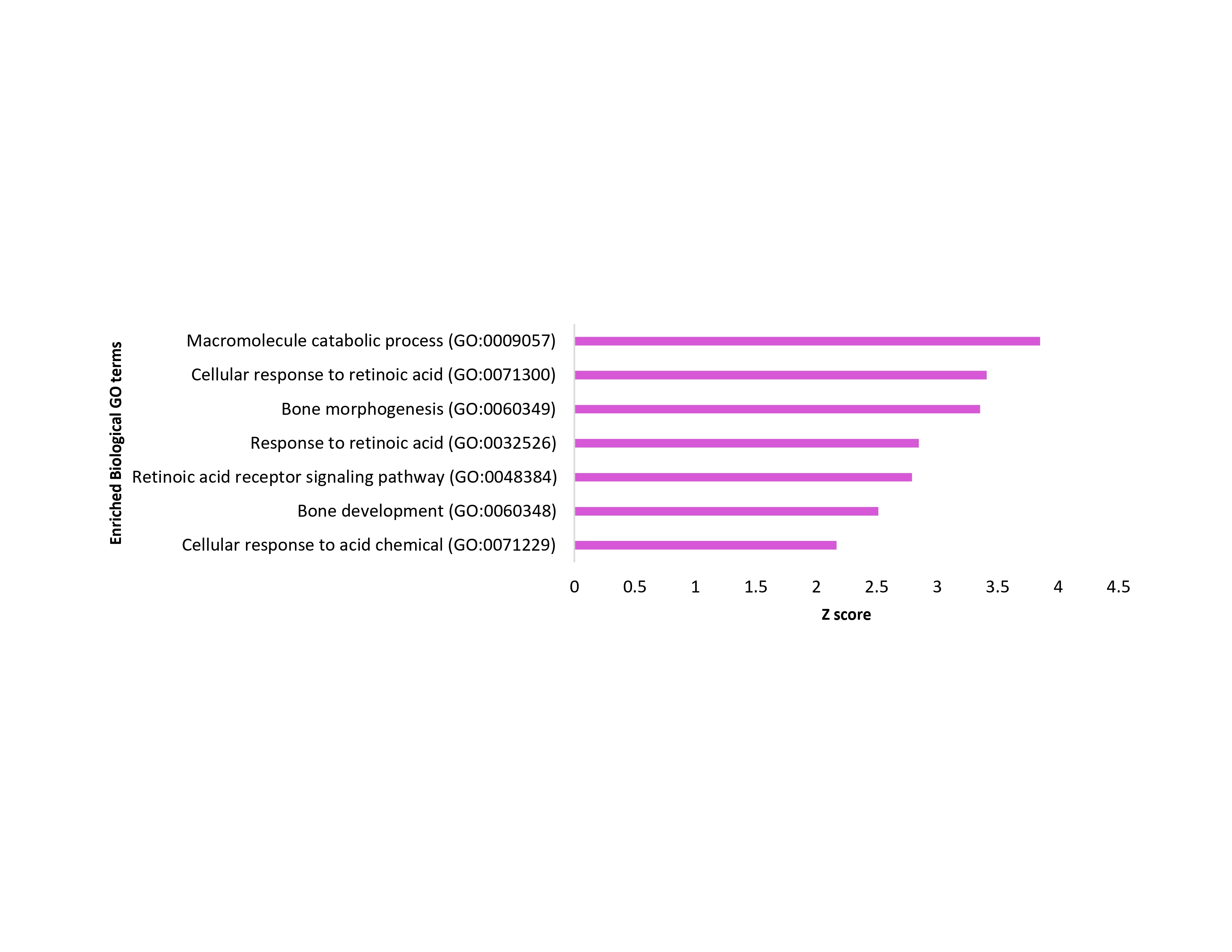

Supplement: Supplementary file 1 [file DataSheet1.ZIP › Supplementry files_3revision/Fig S7a_functional annotation of immunity related genes_Biological process.jpg]

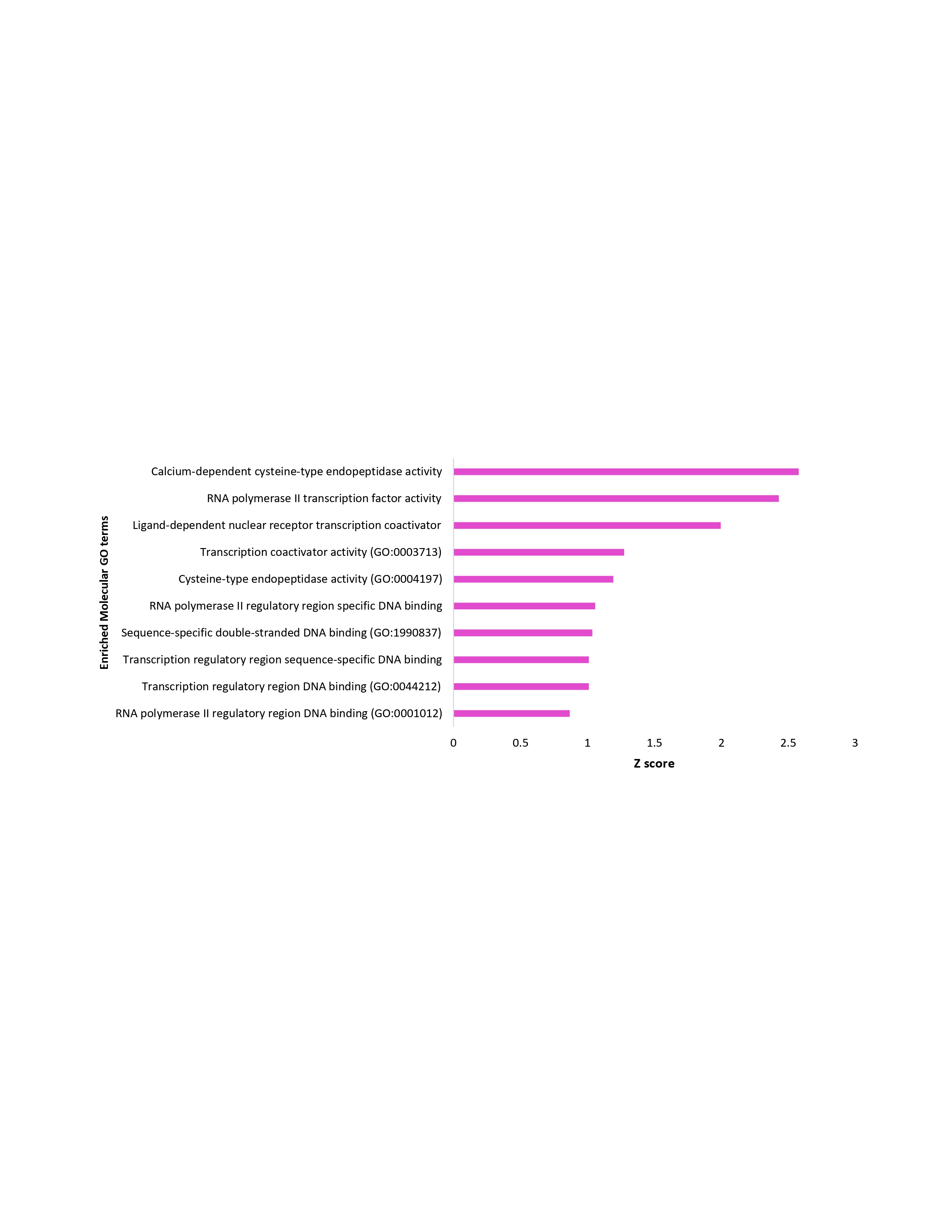

Supplement: Supplementary file 1 [file DataSheet1.ZIP › Supplementry files_3revision/Fig S7b_functional annotation of immunity related genes_Molecular process.jpg]

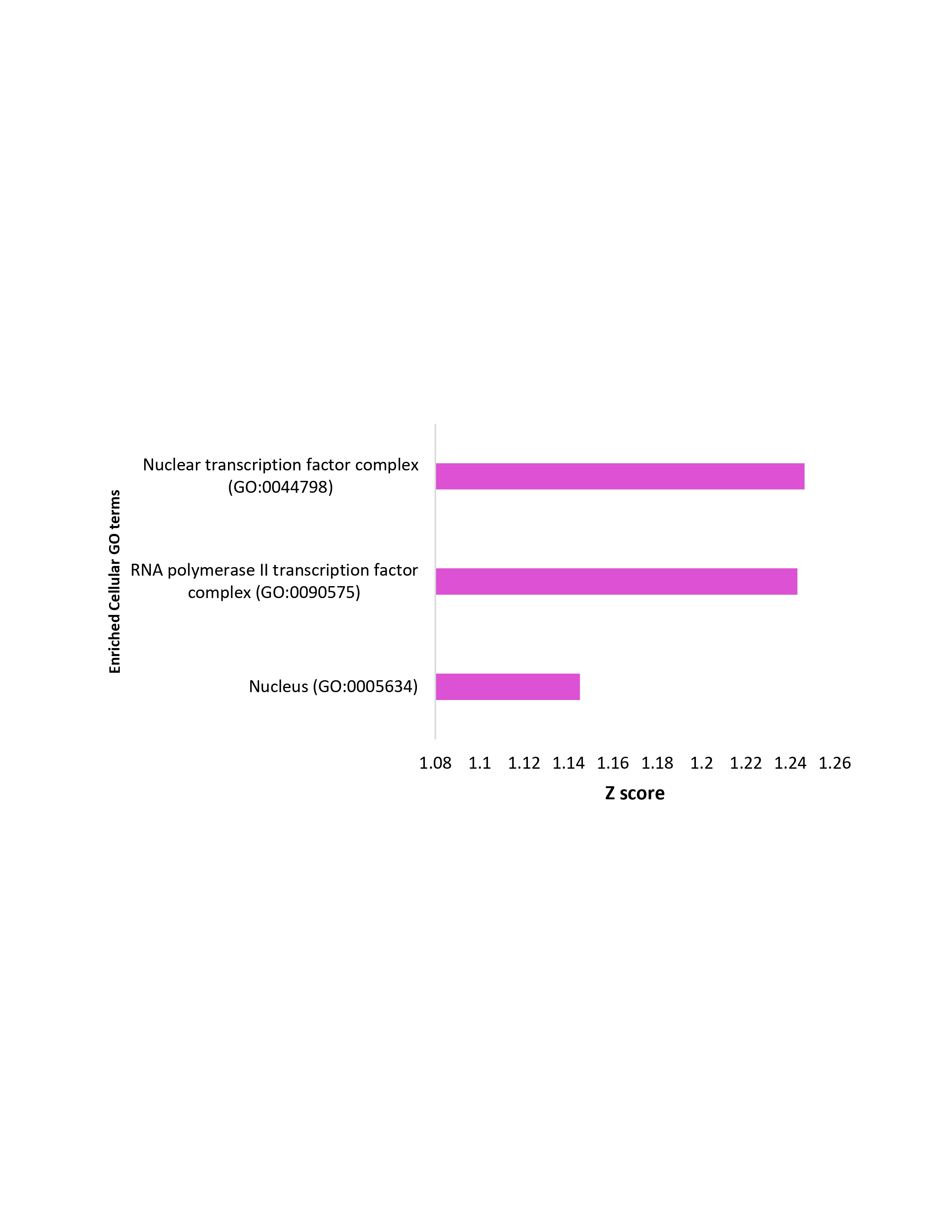

Supplement: Supplementary file 1 [file DataSheet1.ZIP › Supplementry files_3revision/Fig S7C_functional annotation of immunity related genes_Cellular Compartment.jpg]

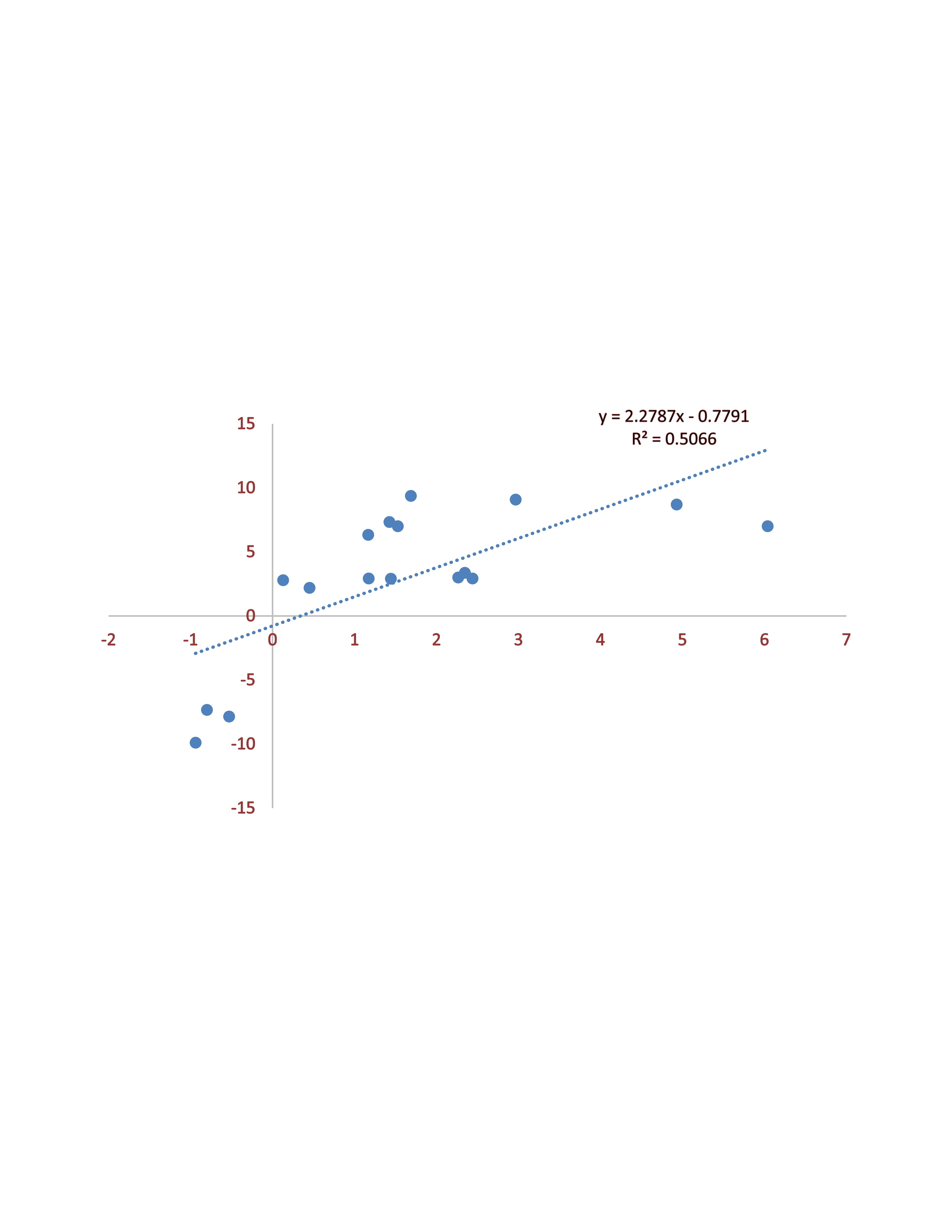

Supplement: Supplementary file 1 [file DataSheet1.ZIP › Supplementry files_3revision/Fig S8_Correlation of gene expression as revealed by qPCR and RNAseq.jpg]

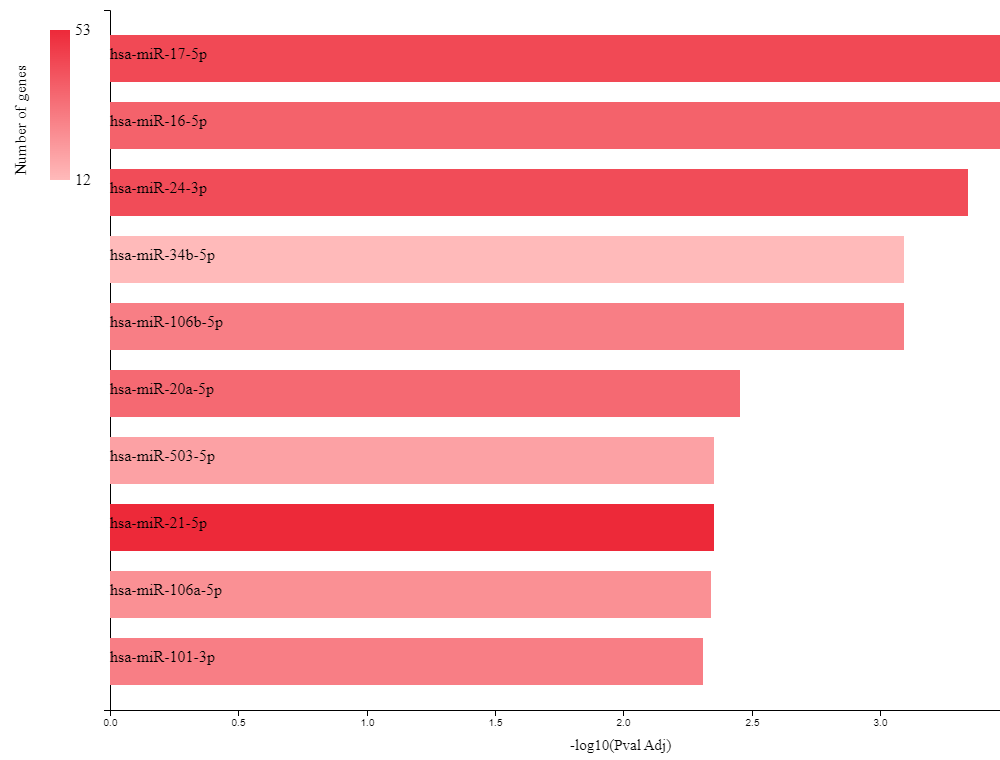

Supplement: Supplementary file 1 [file DataSheet1.ZIP › Supplementry files_3revision/Fig S9 microRNA targets associated with TFs.jpeg]
